# Structure Based Design of Bicyclic Peptide Inhibitors of RbAp48

**DOI:** 10.1002/anie.202009749

**Published:** 2020-11-24

**Authors:** Peter 't Hart, Pascal Hommen, Anaïs Noisier, Adrian Krzyzanowski, Darijan Schüler, Arthur T. Porfetye, Mohammad Akbarzadeh, Ingrid R. Vetter, Hélène Adihou, Herbert Waldmann

**Affiliations:** ^1^ Department of Chemical Biology Max Planck Institute of Molecular Physiology Otto-Hahn-Strasse 11 44227 Dortmund Germany; ^2^ Chemical Genomics Centre of the Max Planck Society Max Planck Institute of Molecular Physiology Otto-Hahn-Strasse 11 44227 Dortmund Germany; ^3^ Medicinal Chemistry, Research and Early Development Cardiovascular Renal and Metabolism, BioPharmaceutical R&D AstraZeneca Gothenburg Sweden; ^4^ Department of Mechanistic Cell Biology Max Planck Institute of Molecular Physiology Otto-Hahn-Strasse 11 44227 Dortmund Germany; ^5^ AstraZeneca MPI Satellite Unit Department of Chemical Biology Max Planck Institute of Molecular Physiology Otto-Hahn-Strasse 11 44227 Dortmund Germany

**Keywords:** cyclization, inhibitors, peptides, protein–protein interactions, structure-based design

## Abstract

The scaffolding protein RbAp48 is part of several epigenetic regulation complexes and is overexpressed in a variety of cancers. In order to develop tool compounds for the study of RbAp48 function, we have developed peptide inhibitors targeting the protein–protein interaction interface between RbAp48 and the scaffold protein MTA1. Based on a MTA1‐derived linear peptide with low micromolar affinity and informed by crystallographic analysis, a bicyclic peptide was developed that inhibits the RbAp48/MTA1 interaction with a very low nanomolar *K*_D_ value of 8.56 nM, and which showed appreciable stability against cellular proteases. Design included exchange of a polar amide cyclization strategy to hydrophobic aromatic linkers enabling mono‐ and bicyclization by means of cysteine alkylation, which improved affinity by direct interaction of the linkers with a hydrophobic residue on RbAp48. Our results demonstrate that stepwise evolution of a structure‐based design is a suitable strategy for inhibitor development targeting PPIs.

## Graphical abstract

Potent bicyclic peptide inhibitors of the RbAp48‐MTA1 interaction were developed by structure based stepwise optimization of the cyclization linker. The strategy exemplifies design of peptide derived inhibitors of protein–protein interactions involving large surface areas.

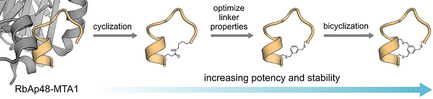

## Introduction

**Figure 1:**
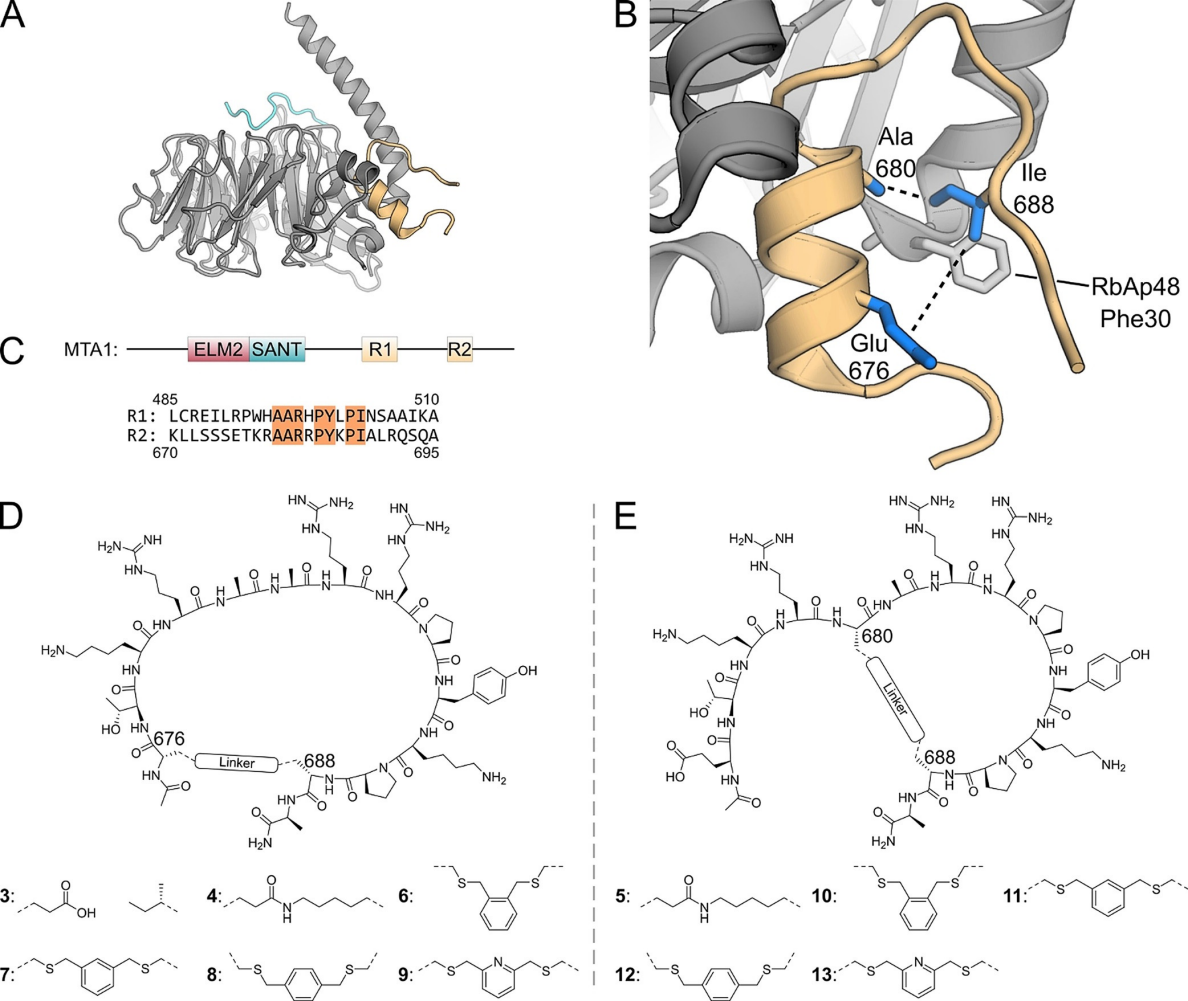
A) RbAp48 with the MTA1 R2 fragment (residues 670–695, orange) bound to the flank binding site. The FOG‐1 peptide (residues 1–13, cyan) is bound to the top site. Superimposition of PDB files 4pbz and 2xu7. B) Zoom of crystal structure of RbAp48 bound by MTA1 R2 peptide (residues 670–695, PDB: 4pbz).^[10]^ Indicated are the peptide positions used for cyclization (blue side chains). C) MTA1 domain structure and sequences of the MTA1 R1 and R2 binding sites. Identical amino acids in both binding sites are highlighted. D) Structure of cyclic peptides **3**, **4**, **6**–**9**. E) Structure of cyclic peptides **5**, **10**–**13**.

RbAp48 (Retinoblastoma‐binding protein 48, also known as RBBP4 or NURF55) is a WD40 repeat containing histone binding protein which is found as a component of a variety of histone modifying complexes including Hat1, NuRD, PRC2, and CAF‐1.^[1]^ As such it plays a role in acetylation, deacetylation and methylation of histones, but also assembly and remodeling of chromatin.[^1a^, ^2^] Overexpression of RbAp48 was found in several cancer types including breast cancer, thyroid carcinomas, hepatocellular carcinoma, colon cancer and models of embryonal brain tumors.^[3]^ The critical role played by RbAp48 makes it an attractive target for modulation of its biological function which may translate into therapeutic intervention. RbAp48 is a member of the WD40 repeat protein family and as such does not have any catalytic function. WD40 proteins typically act as scaffolds for assembly of larger complexes and RbAp48 has two characterized binding sites for protein complex formation (see Figure 1 A).^[1a]^ We hypothesized that protein–protein interaction inhibitors targeting RbAp48 could be invaluable tools to gain further insight into biology and might inspire new medicinal chemistry programs. Similar strategies have proven useful for proteins from the same family such as WDR5 and EED.[^1a^, ^4^]

## Results and Discussion

### The RbAp48‐MTA1 interaction

To inhibit protein–protein interactions involving RbAp48 a structure‐based design approach was chosen with the goal to develop macrocyclic peptide inhibitors.^[5]^ Strong inhibition of protein–protein interactions is often challenging to achieve using small molecules. New modalities such as cyclic peptides are able to cover more surface area and may be better suited to make the required contacts for high affinity binding.[^5a^, ^6^]

Figure 1 A shows RbAp48 in complex with FOG‐1 (cyan) and MTA1 (orange). The FOG‐1 site has been targeted previously using linear peptides with low μM binding affinity.^[7]^ Thus, this binding site has a targetable pocket. However, it is a conserved binding site amongst WD40 repeat proteins which might lead to selectivity issues for potential ligands. In contrast, the flank binding side is unique amongst the WD40 proteins and is therefore a more attractive target (see Figure 1 A/B). The flank binding site is required for interaction with MTA1, Suz12, and H4 and several well‐defined crystal structures of RbAp48 complexes are available.[^2c^, ^8^] MTA1 is a scaffold protein of the NuRD complex and uses its ELM2 and SANT domain to recruit HDAC1/2. It can recruit two copies of RbAp48 using two highly similar binding sites referred to as R1 and R2 (see Figure 1 C).^[9]^ These binding sites have similar sequences and crystallographic information is available (R1: pdb 5fxy; R2: pdb 4pbz).[^8a^, ^10^] Both structures with either the MTA1‐R1 or R2 peptide show a helical section followed by a proline turn and a linear section parallel to the helix (see Figure 1 B). Such a preorganization offered a good starting point for the design of cyclic peptide inhibitors since there were several amino acid side chains facing towards the center of the fold making them suitable for possible cyclization.^[5b]^

Here we report the design, synthesis and evaluation of macrocyclic peptides derived from MTA1 as potent inhibitors of the RbAp48‐MTA1 interaction. Structure based optimization allowed the stepwise increase in potency of the peptides by first optimizing the chemical properties of the cyclization linker followed by converting the monocyclic peptide to a bicyclic variant.

### Design, synthesis and evaluation of R2 derived peptides

The binding affinity of our library of synthetic cyclic peptides was evaluated by a competitive fluorescence polarization (FP) assay using a complex formed by peptide **1** and RbAp48. To reduce the length of the peptide it was truncated to the amino acids which seemed most critical according to the crystal structure (residues 676–689, pdb:4pbz). The 14 amino acid truncated peptide **3** had an IC_50_ of 2.62 μM (see Table 1) which was not unexpected since Alqarni et al. had demonstrated that the MTA1 residues Leu 672, Ile 688 and Leu 690 form a hydrophobic cluster around RbAp48 Phe 30,^[10]^ and two of these had been removed in the truncation. We chose to use a cyclization approach to regain affinity, that is, not to induce more interactions but through minimizing the entropic penalty upon binding by reducing the flexibility of the peptide. To this end two amino acids were chosen which are facing one another in the structure (see Figure 1 B/D/E). Two peptides were designed where glutamic acid was used in position 676 (**4**) or position 680 (**5**) and lysine in position 688 for both peptides (see Table 1). The macrocycles were formed by connecting the side chains via an amide bond (see Figure 1 D/E). Peptide synthesis was performed on Rink‐amide resin using the Fmoc‐strategy incorporating Fmoc‐L‐Glu(All)‐OH and Fmoc‐L‐Lys(Alloc)‐OH at the sites for cyclization. After completion of the linear synthesis the allyl protecting groups were removed using Pd^0^ catalysis and the amide link was formed by using PyBOP. When tested in the competitive FP assay both peptides had improved IC_50_ values of 47.7 nM (**4**) and 125.6 nM (**5**) respectively. Although cyclization significantly improved the IC_50_ values, the newly formed amide potentially is positioned above a hydrophobic patch on RbAp48 formed by Phe 30 and Leu 31. Therefore, a range of peptides was synthesized which were cyclized using the same amino acid positions but via a cysteine alkylation strategy (see Figure 1 D/E and Table 1). In this method the residues used for cyclization are replaced with cysteine and benzylic dibromides act as crosslinkers on the unprotected peptides.^[11]^ Previous research has shown that such linkers can effectively stabilize cyclic peptides and yield high affinity ligands for protein–protein interaction inhibition.^[12]^ Such a linker introduces a xylene group in close vicinity of the hydrophobic patch potentially reconstituting the lost key hydrophobic interactions. To explore a variety of macrocycle conformations we used either ortho‐, meta‐ or para‐substituted dibromides to exchange the linker in both **4** and **5** (see Figure 1 D/E and Table 1).^[13]^ For xylene linker peptides derived from **4** an increase of potency was observed to 18.7 nM and 15.1 nM for **7** and **8** respectively. For those derived from **5**, potency increased to 34.9 nM for peptide **9** (Table 1). The improvements in IC_50_ proved the advantage of introducing a hydrophobic linker for this macrocycle design.

**Table 1:** Structure and IC_50_ values of all R2 sequence derived peptides. For a full overview of compound structures and IC_50_ values see supplemental Table 2.

Peptide^[a]^	Sequence/mutation^[b]^	Cyclization	IC_50_ [nM]
**1**	**676 680 688** FITC‐PEG‐KLLSSS**E**TKR**A**ARRPYKP**I**ALRQSQA‐NH_2_	–	—
**2**	Ac‐KLLSSSETKRAARRPYKPIALRQSQA‐NH_2_	–	13.4±3.0
**3**	Ac‐ETKRAARRPYKPIA‐NH_2_	–	2621±786
**4**	Ac‐**E**TKRAARRPYKP**K**A‐NH_2_	amide	47.7±12.5
**5**	Ac‐ETKR**E**ARRPYKP**K**A‐NH_2_	amide	125.6±32.3
**6**	Ac‐**C**TKRAARRPYKP**C**A‐NH_2_	ortho xylene	77.4±6.5
**7**	Ac‐**C**TKRAARRPYKP**C**A‐NH_2_	meta xylene	18.7±4.1
**8**	Ac‐**C**TKRAARRPYKP**C**A‐NH_2_	para xylene	15.1±5.4
**9**	Ac‐**C**TKRAARRPYKP**C**A‐NH_2_	meta pyridine	34.9±14.0
**10**	Ac‐ETKR**C**ARRPYKP**C**A‐NH_2_	ortho xylene	321.7±56.8
**11**	Ac‐ETKR**C**ARRPYKP**C**A‐NH_2_	meta xylene	388.0±69.8
**12**	Ac‐ETKR**C**ARRPYKP**C**A‐NH_2_	para xylene	2342±363
**13**	Ac‐ETKR**C**ARRPYKP**C**A‐NH_2_	meta pyridine	146.8±10.7
**33**	Ac‐**C**TKR**C**ARRPYKP**C**A‐NH_2_	mesitylene	12.3±2.0
**36** (R2 scrambled)	Ac‐**C**PRA**C**RYKTAPR**C**K‐NH_2_	mesitylene	>10 000
**37**	Ac‐**C****A**KR**C**ARRPYKP**C**A‐NH_2_	mesitylene	12.4±3.2
**38**	Ac‐**C**T**A**R**C**ARRPYKP**C**A‐NH_2_	mesitylene	262.9±42.7
**39**	Ac‐**C**TK**A****C**ARRPYKP**C**A‐NH_2_	mesitylene	77.4±9.6
**40**	Ac‐**C**TKR**C**A**A**RPYKP**C**A‐NH_2_	mesitylene	4319±974
**41**	Ac‐**C**TKR**C**AR**A**PYKP**C**A‐NH_2_	mesitylene	24.6±3.9
**42**	Ac‐**C**TKR**C**ARR**A**YKP**C**A‐NH_2_	mesitylene	131.3±15.3
**43**	Ac‐**C**TKR**C**ARRP**A**KP**C**A‐NH_2_	mesitylene	38.3±1.9
**44**	Ac‐**C**TKR**C**ARRPY**A**P**C**A‐NH_2_	mesitylene	19.8±3.6
**45**	Ac‐**C**TKR**C**ARRPYK**A****C**A‐NH_2_	mesitylene	15.6±2.2
**46**	Ac‐**C**TKR**C**ARRPYKP**C**‐NH_2_	mesitylene	20.5±1.4
**47**	N_3_‐PEG‐**C**TKR**C**ARRPYKP**C**A‐NH_2_	mesitylene	13.9±5.6
**48** (R2 scrambled)	N_3_‐PEG‐**C**PRA**C**RYKTAPR**C**K‐NH_2_	mesitylene	>10 000
**49** (Tat‐modified)	H‐GRKKRRQRRRPQG**C**TKR**C**ARRPYKPCA‐NH_2_	mesitylene	4.4±1.4
**50**	H‐GRKKRRQRRRPQ‐NH_2_	–	1239±358

[a] All sequences are derived from the MTA1‐R2 sequence unless otherwise indicated. [b] Residues used for cyclization are highlighted in blue. Mutated residues are highlighted in red.

### Design, synthesis and evaluation of R1 derived peptides

Compound design described above had only focused on the MTA1‐R2 sequence, and for further improvement, the MTA‐R1 sequence was also explored (see Figure 1 C, peptides **14**–**19** in supplemental Table 2). Since larger macrocycles were better for the R2 sequence as described above, a similar design was employed for the R1‐sequence (see supplemental Table 2 and supplemental Figure 2). To identify the best N‐terminal connection point both position 490 and 491 were explored, and both linear sequences **14** and **15** were synthesized and cyclic variants were prepared through cysteine alkylation (**16**–**19**). When tested in the competitive FP assay, neither of these peptides showed appreciable IC_50_ values. To further investigate the roles of specific amino acids in the R1 and R2 sequences, hybrid peptides were prepared where the cyclic R2 sequence was used as a basis and point mutations using amino acids from the R1 sequence were introduced (peptides **20**–**27,** see supplemental Table 2). Most peptides were poorly active except for **20** and **21** which had IC_50_ values similar to those of **7** and **8** (see supplemental Table 2).

### X‐ray analysis of RbAp48 bound to peptide 8

**Figure 2:**
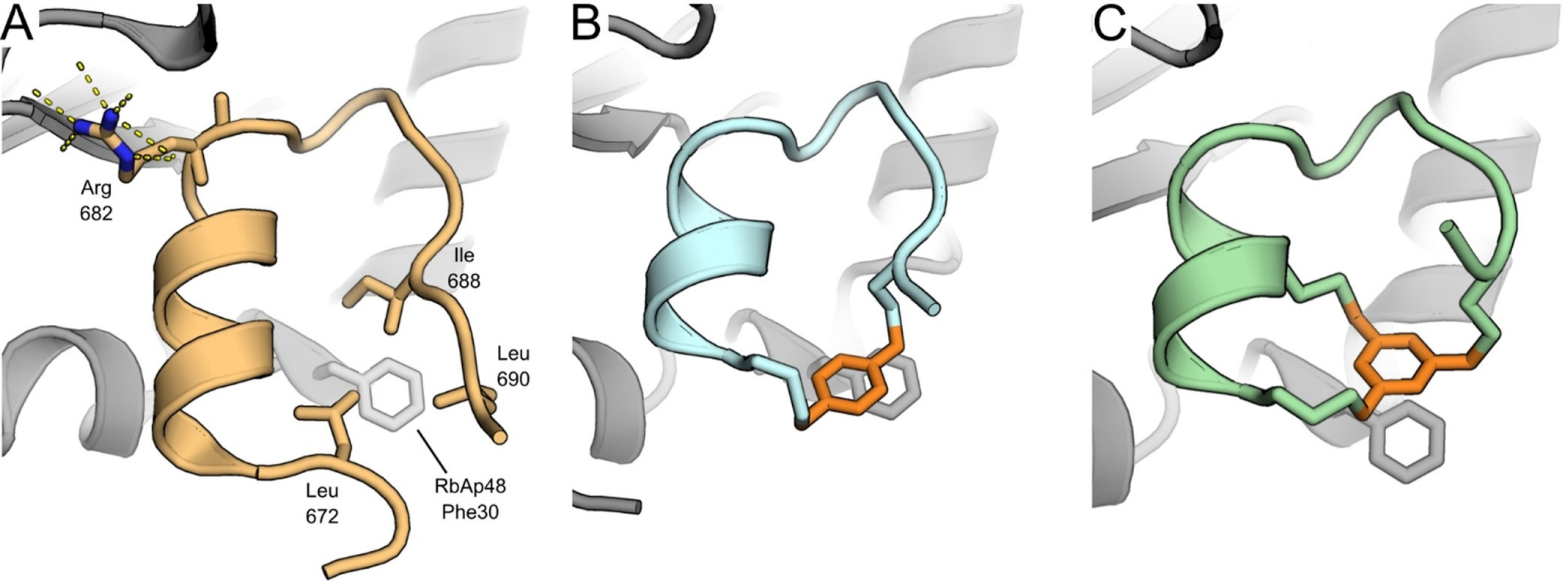
A) Crystal structure of MTA1 R2 peptide bound to RbAp48 (PDB: 4pbz).^[10]^ Shown are the three amino acids forming the hydrophobic cluster around Phe 30 of RbAp48 and the hydrogen bonding network of Arg 682. B) Peptide **8** bound to RbAp48. The xylene linker is highlighted in orange. (PDB: 6ZRC) C) Peptide **33** bound to RbAp48. The mesitylene linker is highlighted in orange. (PDB: 6ZRD).

Encouraged by these results we investigated the molecular details of the interaction between these peptides and RbAp48 by X‐ray analysis of cocrystals. The cocrystal structure of peptide **8** with RbAp48 revealed that the xylene linker was in close proximity to Phe 30 on RbAp48 similar to the hydrophobic cluster of the original R2 peptide (see Figure 2 A/B). The crystal structure confirmed our hypothesis of the advantage of a hydrophobic linker over an amide linker and indicated a possible π‐stacking interaction. We tried to take advantage of the potential π‐stacking by preparing a variety of substituted linkers which would modulate the interaction by either having electron withdrawing or electron donating effects on xylene linker (compounds **28**–**32**, see supplemental Figure 2 and supplemental Table 2). Surprisingly, all modifications led to a 1.5–2.5 fold reduction in potency, potentially indicating that the interaction is driven by van der Waals forces rather than π‐π interactions and bulkier substitutions are not tolerated.

### Design, synthesis and evaluation of bicyclic peptides

**Figure 3:**
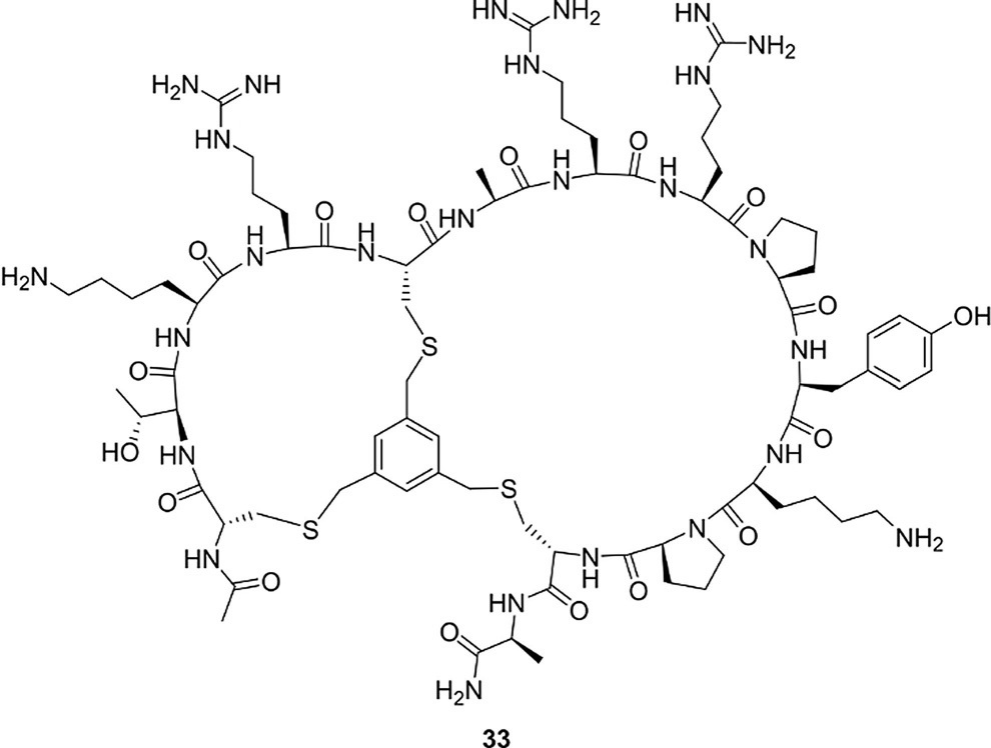
Structure of compound **33**.

Larger macrocyclic peptides (>10 amino acids) may suffer from proteolytic instability and a relatively low affinity due to their high flexibility.^[14]^ It has therefore been proposed that bicyclization can further optimize these properties by reducing the size of the macrocycle and therefore constraining the peptide further.[^12a^, ^15^] The crystal structural of **8** bound to RbAp48 indicated that the side chain of Ala 680 was pointing towards the cyclization linker and could be explored as an option for the design of bicyclic peptides (see Figure 1 B and 3 B). Traditionally, hydrocarbon stapling has been used to stabilize α‐helices.^[16]^ Other well‐known strategies include the N‐terminal capping method which mimics hydrogen bonds and induces helix formation.^[17]^ Complementary to these approaches, the cysteine alkylation strategy has previously been used to stabilize α‐helical structures and the m‐xylene linker was found to be most optimal.^[18]^ Following these principles a bicyclic peptide was designed by introducing cysteine residues in all three cyclization positions (see Figure 3 and Table 1) and reacting it with 1,3,5‐tris(bromomethyl)benzene.[^11a^, ^19^] The strategy stabilizes the large macrocycle and the N‐terminal α‐helix at the same time to impose a high constraint on peptide flexibility. In the FP assay the R2 derived peptide **33** showed potent binding affinity (12.3 nM, see Table 1), while the R1 derived peptides **34** and **35** were much less potent (152.9 and 7485 nM respectively, see supplemental Table 2). Cocrystallization of RbAp48 with **33** confirmed that the bicyclization strategy successfully stabilized the peptide fold (see Figure 2 C). Furthermore, the mesitylene linker was again observed in close proximity to Phe 30 on RbAp48. To evaluate the structure activity relationship the peptide was subjected to scrambling (**36**), alanine scanning (**37**–**45**), and truncation of the exocyclic terminal alanine (**46**, see Table 1). The IC_50_ values determined for the series indicated that scrambling caused the peptide to completely lose affinity. In the alanine scanning series each residue was mutated to alanine once and it was found that Arg 682 (mutated in peptide **40**) was critically important for the interaction, since the potency of the corresponding mutated peptide **40** decreased several orders of magnitude (Table 1, compare entries **33** and **40**)The importance of this residue is illustrated by the extensive hydrogen bonding network made by the guanidine with several residues on RbAp48 (see Figure 2 A). Furthermore, the arginine is conserved in both the R1 and R2 binding sites. Mutation of Lys 678, Arg 679 and Pro 684 led to a 6–20 fold decrease in activity while other mutations had minor effects on binding affinity (Table 1, compare entries **38**, **39**, **42** to **33**). All other modifications did not have a significant effect on the potency.

### Further biophysical analysis of key peptides

**Figure 4:**
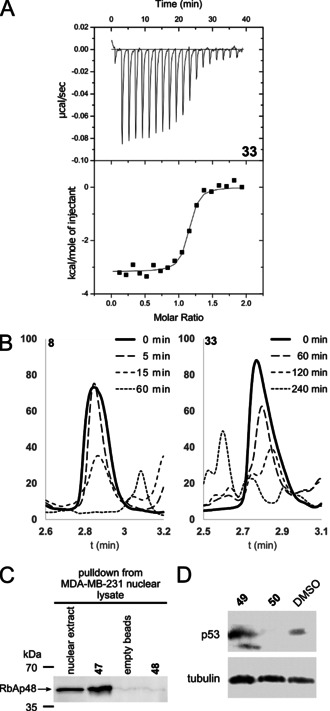
A) Representative thermogram of titration of compound **33** into RbAp48 + compound **40**. B) HPLC analysis of degradation of compound **8** and **33** in MDA‐MB‐231 cell lysate. C) Pulldown assay with peptides **47** and **48** immobilized on DBCO beads. RbAp48 was pulled down from nuclear lysate of MDA‐MB‐231 cells. D) U2OS cells were treated with compound **49**, **50**, or DMSO and analyzed by western blot for levels of p53.

Since under the conditions of the employed competitive FP assay IC_50_ values can only be determined to the protein concentration employed (15 nM), we analysed the compounds additionally by means of competitive isothermal titration calorimetry. In this method the cell is loaded with protein and a weak binding ligand (**40**, *K*_D_=15.8 μM, see Table 2).^[20]^ The weak ligand interferes with binding of the strong ligand allowing the use of higher concentrations and therefore stronger signal. The actual affinity and thermodynamic parameters can then be calculated from the measured values.[^20^, ^21^] The long linear peptide **2**, monocyclic peptide **8**, and bicyclic peptide **33** were analysed using this method and the results are reported in Table 2 (See Figure 4 A and Supplemental Figure 13–16). The *K*_D_ observed for **2** was 5.18 nM which was similar to bicyclic peptide **33** which showed a *K*_D_ value of 8.56 nM. Under the same conditions monocyclic peptide **8** did not yield a binding curve indicating it has a lower affinity than both **2** and **33** which further confirms that bicyclization is advantageous. An effect which could not clearly be observed by the FP assay as it's lower limits were reached.

**Table 2:** Thermodynamic parameters of compounds **2**, **8**, **33** and **40** measured by ITC.

Peptide ^[a]^	*K*_D_ [nM]	Δ*G* [kcal mol^−1^]	Δ*H* [kcal mol^−1^]	−*T*Δ*S* [kcal mol^−1^]
**2** ^[a]^	5.18±0.54	−11.31±0.06	−11.03±0.13	−0.28±0.19
**8** ^[a]^	N.B.	–	–	–
**33** ^[a]^	8.56±5.83	−11.08±0.44	−7.10±0.02	−3.98±0.42
**40** ^[b]^	15.8×10^3^±1.2×10^3^	−6.55±0.04	−4.10±0.13	−2.45±0.09

[a] Measured by titrating 300 μM peptide into a cell containing 30 μM RbAp48 and 416 μM peptide **40**. [b] Measured by titrating 800 μM peptide into a cell containing 40 μM RbAp48. N.B.: no binding under the used conditions.

Circular dichroism (CD) spectroscopy analysis of the linear peptides **2** and **3**, monocyclic peptides **4** and **7**, and the bicyclic **33** was performed to evaluate whether structural preorganization correlated with binding affinity. The CD measurement indicated the peptides do not have an obvious structural preorganization in solution as all spectra are indicative of a random coil (see supplemental Figure 17).

To investigate whether the cyclization strategy influences the stability of the peptides we incubated **3**, **8** and **33** in MDA‐MB‐231 cell lysate. HPLC analysis indicated rapid degradation for both the linear (**3**, *t*_1/2_=16.0 min) and the monocyclic peptide (**8**, *t*_1/2_=11.9 min) (see Figure 4 B and supplemental Figures 18–20). The bicyclic peptide **33** was significantly more stable with a half‐life of 94.3 minutes.

### Biological evaluation of peptide 33 and derivatives

To evaluate whether peptide **33** interacts with RbAp48 in a more complex biological environment we modified the N‐terminus with a linker bearing a terminal azide (**47,** see Table 1, supplemental Figure 4). The peptides were immobilized on DBCO‐beads via copper‐free click chemistry and exposed to MDA‐MB‐231 nuclear lysate. As a negative control an azide modified scrambled peptide sequence (**48**) with affinity >10 μM was used. As shown in Figure 4 C compound **47** was successfully able to enrich RbAp48 while the negative probe (**48**) showed no interaction.

**Figure 5:**
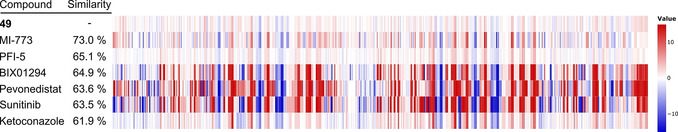
Cell painting fingerprint comparison of compound **49** and reference compounds relating to p53 induction or stabilization.

Encouraged by the promising results from the pulldown experiment, and in order to get insight into bioactivity and possible mode of action, we analyzed peptide **33** in a morphological cell painting assay. The cell painting assay is a target agnostic assay which determines a cells morphological profile after treatment with a compound of interest and compares it to the profiles of a set of reference compounds.^[22]^ By identifying reference compounds with a similar profile a hypothesis of the compounds mode of action can be generated based on their annotated targets.^[23]^ U2OS cells were treated with our compound and 579 parameters were analyzed. For each compound tested an induction score is calculated which reflects the number of parameters which have changed significantly compared to the DMSO control.^[23b]^ Initially compound **33** showed no induction which we hypothesized was due to a low membrane permeability. We therefore synthesized a variant modified with an N‐terminal TAT sequence to enhance its permeability (compound **49**, see Table 1 and Supplemental Figure 4).^[24]^ Compound **49** was first tested in the FP assay and found to be slightly more active than its parent compound **33**. The TAT sequence alone (compound **50**, see Table 1) was found to weakly inhibit the interaction in the FP assay albeit at an IC_50_ three orders of magnitude higher than **49**. Applying compound **49** to the cell painting assay led to an induction of 5.9 % and the corresponding profile was compared to the set of 3000 reference compounds. Several of the compounds with highest similarity in the bioactivity profile increase levels of the tumor suppressor protein p53 (see Figure 5 and supplemental Table 6)^[25]^ which suggests that peptide **49** might share this activity. To validate this hypothesis, we exposed U2OS cells to compound **49** and evaluated the levels of p53 by Western blot. The results indicate that p53 is indeed increased upon treatment with compound **49** and no increase is observed when the cells are exposed to TAT peptide **50** alone (See Figure 4 D). The exact mechanism by which the compounds elevate levels of p53 requires further investigation. However, it has previously been shown that the NuRD complex, of which RbAp48 is a core component, is able to deacetylate p53 and thereby modulate its stability.^[26]^ The effect is mediated by the MTA family of proteins from which the compounds described here are derived.

## Conclusion

In conclusion, following the goal to develop tool compounds that may enable further studies of the biological programs modulated by RbAp48, we have developed potent bicyclic peptide inhibitors via a structure‐based approach. To this end we focused on the interaction of RbAp48 with MTA1 and selected a peptide sequence derived from MTA1 as starting point for inhibitor development. Structure‐based design led to the identification of a bicyclic peptide (**33**) that inhibits the RbAp48/MTA1 interaction with a very low nanomolar *K*_D_ value of 8.56 nM, and which also showed appreciable stability against cellular proteases. Conversion of compound **33** to the cell permeable **49** allowed the evaluation of the mode of action using a cell painting assay. This indicated the compounds mode of action could be related to p53 induction or stabilization. The hypothesis was confirmed by treatment of U2OS cells with compound 49 followed by evaluation of the p53 protein levels. Thus, peptide **49** may be employed as tool for subsequent biological studies. The majority of bicyclic peptides is obtained from either synthetic or biologically generated combinatorial libraries.[^1^, ^2^, ^3^, ^4^] The work described here shows that peptide bicyclization using cysteine alkylation to stepwise evolve a structure‐based design is suitable for PPIs indicating that this strategy may be successfully applied in further cases.

## Conflict of interest

The authors declare no conflict of interest.

## Supporting information

As a service to our authors and readers, this journal provides supporting information supplied by the authors. Such materials are peer reviewed and may be re‐organized for online delivery, but are not copy‐edited or typeset. Technical support issues arising from supporting information (other than missing files) should be addressed to the authors.

Supplementary
