# Supplementary material for: Structure Based Design of Bicyclic Peptide Inhibitors of RbAp48
Source: Angew Chem Int Ed Engl. 2020 Nov 24;60(4):1813–20. doi: 10.1002/anie.202009749 (PMC7894522; doi:10.1002/anie.202009749)
Supplement: Supplementary file 1 — Supplementary [file ANIE-60-1813-s001.pdf]

Angewandte Chemie
Eine Zeitschrift der Gesellschaft Deutscher Chemiker

Supporting Information

Structure Based Design of Bicyclic Peptide Inhibitors of RbAp48

*Peter 't Hart, Pascal Hommen, Anaïs Noisier, Adrian Krzyzanowski, Darijan Schüler, Arthur T. Porfetye, Mohammad Akbarzadeh, Ingrid R. Vetter, Hélène Adihou, and Herbert Waldmann**

anie_202009749_sm_miscellaneous_information.pdf

Contents

Synthetic methods ... 2
- Method A (linear peptide synthesis): ... 2
- Method B (linear peptide synthesis): ... 2
- Method C (cyclization via amide bond formation): ... 2
- Method D (Cyclization via cysteine alkylation): ... 2
- Method E (Cyclization via cysteine alkylation): ... 2
- Fluorescently labelled peptides ... 2

Peptide purification ... 3
    - Method A: ... 3
    - Method B: ... 3
    - Method C: ... 3
- Synthesis/purification methods for all peptides and HRMS data ... 3

Protein expression and purification ... 5

Fluorescence polarization assay ... 5

Isothermal Titration Calorimetry ... 5

Circular dichroism ... 5

Stability assay ... 5

Pull-down experiments ... 6

Cell painting assay ... 6

p53 expression level assay ... 8

Overview of compounds ... 10

Fluorescence polarization results ... 14

ITC thermograms ... 20

Circular dichroism spectra ... 22

Peptide stability in cell lysate ... 23

References ... 26

HPLC Analysis of compounds **1** – **50** ... 27
- Analytical method A: ... 27
- Analytical method B: ... 27
- Analytical method C: ... 27

Crystallization and structure determination ... 42

Synthetic methods

Peptides were synthesized according to one of the following methods:

Method A (linear peptide synthesis):

Synthesis was performed on Rink Amide MBHA resin (0.4 mmol/g) using an automated CEM Librety microwave peptide synthesizer. Amino acid coupling reactions were done at 75°C (40 W) for 5 minutes using 5 eq amino acid, 5 eq PyBOP and 10 eq DIPEA in DMF. Fmoc-deprotection was performed by addition of 20% piperidine in DMF and reaction at 75°C (40 W) for 30 seconds followed by addition of fresh reagents and reaction at 75°C (40 W) for 3 minutes. Peptides were cleaved using TFA/ODT/TIPS/H_2O (90/2.5/2.5/5) for 2 x 1 h or for 1 h per arginine in the sequence. Peptides were precipitated in cold Et_2O followed by centrifugation. The supernatant was removed and the pellet resuspended in fresh cold Et_2O, the procedure was repeated once more. The crude product was dissolved in H_2O/ACN (1:1) and lyophilized.

Method B (linear peptide synthesis):

Synthesis was performed on Rink Amide MBHA resin (0.61 mmol/g) using an automated Syro II parallel peptide synthesizer. Amino acid coupling reactions were done at rt for 40 minutes using 4 eq amino acid, 4 eq HATU and 8 eq DIPEA in DMF. All amino acids were double coupled. Fmoc-deprotection was performed by addition of 40% piperidine in DMF for 3 min followed by addition of fresh reagents and reaction for 10 minutes. Peptides were cleaved using TFA/TIPS/H_2O (90/2.5/2.5) for 2 h. Peptides were precipitated in cold Et_2O followed by centrifugation. The crude product was dissolved in H_2O/ACN (1:1) and lyophilized.

Method C (cyclization via amide bond formation):

The amino acids to be cyclized were introduced as Fmoc-Glu(All)-OH and Fmoc-Lys(Alloc)-OH. After linear synthesis, using the same method as described for linear peptides, the allyl protecting groups were removed by treating the resin with $Pd(PPh_3)_4$ (0.25 eq) and $PhSiH_3$ (25 eq) in dry DCM for 1 h. The liquid was removed and fresh reagents were added and reacted for another hour. The resin was washed with DCM (4x), DMF (2x), 0.5% diethyldithiocarbamic acid trihydrate sodium salt in DMF (4x) and DMF (4x). Next the resin was treated with PyBOP (4 eq) and DIPEA (8 eq) in DMF for 2 h followed by peptide cleavage and purified following the same protocol as for linear peptides.

Method D (Cyclization via cysteine alkylation):

Lyophilized linear peptides were dissolved in 20 mM NH_4HCO_3/ACN (3:1) and 1.1 eq of the appropriate bromide reagent was added as a solution in a small amount of ACN. After 1 h the reaction mixture was lyophilized and peptides were purified as described below.

Method E (Cyclization via cysteine alkylation):

To a 0.33 mM solution of crude peptide dissolved in a 1:1 solution of H_2O/MeCN, the core (1.2 eq) predissolved in a minimum amount of MeCN was added, followed by a solution of 60 mM NH_4HCO_3 (to afford a final peptide concentration of 0.5 mM). The resulting clear reaction mixture was stirred at rt for 1h, and freeze dried to afford the crude peptide as a white fluffy solid.

Fluorescently labelled peptides

After linear synthesis, as described above, the peptides were manually modified with Fmoc-O2Oc-OH (4 eq), PyBOP (4 eq) and DIPEA (8 eq) in DMF. Next Fmoc-deprotection was done by treating the resin with 20% piperidine in DMF for 5 min and once more for 10 min. Next, the peptides were treated with FITC (4 eq) and DIPEA (8 eq) twice for 60 min. Cleavage and purification was similar as described above.

Peptide purification

Method A:

Peptides were purified using a preparative scale C18 column (Macherey-Nagel, 5µM, 125 x 21 mm) at a flow rate of 20 ml/min. The peptides were eluted with a binary mixture of H_2O and ACN, both containing 0.1% TFA, using a linear gradient of 5-50% ACN over 50 min.

Method B:

Similar to method A but MeOH was used instead of ACN.

Method C:

Peptides were purified using a preparative scale reverse-phase column (Atlantis T3 OBD column, 5µM, 150 x 19 mm) at a flow rate of 30 ml/min. The peptides were eluted with a binary mixture of buffer A (H_2O + 0.15% TFA) and buffer B (CAN). Gradients used were determined by elution profiles obtained from analytical RP-UPLC:

Gradient	step 1	step 2	step 3
I	5% B for 1 min	5-22% B in 3 min	22-27% in 15 min
II	5% B for 1 min	5-19% B in 3 min	19-24% in 15 min
III	5% B for 1 min	5-21% B in 3 min	21-26% in 15 min
IV	5% B for 1 min	5-23% B in 3 min	23-28% in 15 min
V	5% B for 1 min	5-10% B in 1.5 min	10-25% in 14 min
VI	5% B for 1 min	5-20% B in 3 min	20-25% in 15 min
VII	5% B for 1 min	5-25% B in 15 min	

Synthesis/purification methods for all peptides and HRMS data

Supplemental table 1: Synthesis and purification methods and HRMS results for peptides **1**-**50**.

Compound	Linear synthesis	Cyclization	Purification	Calculated m/z	Measured m/z
**1**	A	-	A	1744.9152 $[M+H]^+$	1744.9192
**2**	A	-	A	1498.8656 $[M+H]^+$	1498.8692
**3**	A	-	B	849.5003 $[M+2H]^{2+}$	849.4979
**4**	A	C	B	848.0004 $[M+2H]^{2+}$	848.0009
**5**	A	C	B	877.0032 $[M+2H]^{2+}$	877.0010
**6**	A	D	A	882.4696 $[M+2H]^{2+}$	882.4719
**7**	A	D	A	882.4696 $[M+2H]^{2+}$	882.4719
**8**	A	D	A	882.4696 $[M+2H]^{2+}$	882.4711
**9**	A	D	A	882.9672 $[M+2H]^{2+}$	882.9667
**10**	A	D	A	911.4724 $[M+2H]^{2+}$	911.4742
**11**	A	D	A	911.4724 $[M+2H]^{2+}$	911.4733
**12**	A	D	A	911.4724 $[M+2H]^{2+}$	911.4699
**13**	A	D	A	911.9700 $[M+2H]^{2+}$	911.9702
**14**	B	E	C-I	941.5225 $[M+2H]^{2+}$	941.5252
**15**	B	E	C-II	884.9795 $[M+2H]^{2+}$	884.9819
**16**	B	E	C-III	982.4701 $[M+2H]^{2+}$	982.4746
**17**	B	E	C-III	982.4701 $[M+2H]^{2+}$	982.4738
**18**	B	E	C-IV	1807.8313 $[M+H]^+$	1807.8359
**19**	B	E	C-IV	904.4196 $[M+2H]^{2+}$	904.4215
**20**	B	E	C-V	587.3174 $[M+3H]^{3+}$	587.3195
**21**	B	E	C-V	587.3174 $[M+3H]^{3+}$	587.3190

**22**	B	E	C-VI	607.9771 $[M+3H]^{3+}$	607.9797
**23**	B	E	C-II	607.9771 $[M+3H]^{3+}$	607.9762
**24**	B	E	C-V	872.9485 $[M+2H]^{2+}$	872.9511
**25**	B	E	C-V	872.9485 $[M+2H]^{2+}$	872.9508
**26**	B	E	C-V	872.9485 $[M+2H]^{2+}$	872.9522
**27**	B	E	C-V	872.9485 $[M+2H]^{2+}$	872.9506
**28**	B	E	C-VII	598.6525 $[M+3H]^{3+}$	598.6545
**29**	B	E	C-VII	596.9808 $[M+3H]^{3+}$	596.9829
**30**	B	E	C-VII	614.6192 $[M+3H]^{3+}$	614.6205
**31**	B	E	C-VII	593.3209 $[M+3H]^{3+}$	593.3217
**32**	B	E	C-VII	605.3209 $[M+3H]^{3+}$	605.3215
**33**	A	D	A	1807.9035 $[M+H]^{+}$	1807.9075
**34**	B	E	C-VI	1004.4562 $[M+2H]^{2+}$	1004.4608
**35**	B	E	C-I	926.4056 $[M+2H]^{2+}$	926.4076
**36**	A	D	A	603.3064 $[M+3H]^{3+}$	603.3081
**37**	A	D	A	889.4504 $[M+2H]^{2+}$	889.4513
**38**	A	D	A	875.9267 $[M+2H]^{2+}$	875.9279
**39**	A	D	A	861.9236 $[M+2H]^{2+}$	861.9241
**40**	A	D	A	861.9236 $[M+2H]^{2+}$	861.9242
**41**	A	D	A	861.9236 $[M+2H]^{2+}$	861.9237
**42**	A	D	A	891.4478 $[M+2H]^{2+}$	891.4484
**43**	A	D	A	858.4425 $[M+2H]^{2+}$	858.4432
**44**	A	D	A	875.9267 $[M+2H]^{2+}$	875.9274
**45**	A	D	A	891.4478 $[M+2H]^{2+}$	891.4489
**46**	A	D	A	868.9371 $[M+2H]^{2+}$	868.9378
**47**	A	D	A	660.9997 $[M+3H]^{3+}$	661.0010
**48**	A	D	A	660.9997 $[M+3H]^{3+}$	661.0019
**49**	A	D	A	685.9867 $[M+5H]^{5+}$	685.9877
**50**	A	-	A	541.0127 $[M+3H]^{3+}$	541.0119

Protein expression and purification

RbAp48 was expressed and purified by the Dortmund Protein Facility. Full length RbAp48 was cloned into a pOPIN vector ligating it to an N-terminal His tag followed by a 3C cleavage site. The protein was expressed from Sf9 cells for 48 h. Cells were harvested and lysed and the protein was purified by loading onto a HisTrap FF crude 5 ml column followed by on-column cleavage using Precission protease. The cleaved protein was then further purified using a HiLoad 26/60 Superdex 200 column.

Fluorescence polarization assay

Fluorescence polarization assays were performed in 384 wells plates in 80 µl volumes. The assay buffer contains 20 mM Tris-HCl, 150 mM NaCl, 0.01% Tween-20 at pH 7.5. The concentration of the fluorescently labelled peptides was 1 nM plates were incubated at room temperature for 30 min before they were read on a Tecan Spark plate reader (λ_{ex} = 485 nm, λ_{em} = 535 nm).

For competitive assays the same conditions were used with a tracer concentration of 1 nM was used and a protein concentration of 15 nM.

Isothermal Titration Calorimetry

Isothermal titration calorimetry measurements were performed using a Microcal ITC-200 device. The buffer for all measurements consisted of 20 mM Tris, 150 mM NaCl, pH 7.5 and all measurements were performed at 25°C in duplicate except for **8**. RbAp48 was dialyzed overnight using a 10 kDa MWCO Slide-A-lizer dialysis membrane and peptide samples were dissolved in the dialysis buffer. Direct measurements of **40** were perfomed by titrating the peptide (800 µM) into the cell containing RbAp48 (40 µM) using 2 µl injection after an initial injection of 0.5 µl.

Direct titration of **2** and **33** gave curves which were hard to fit and therefore their affinity was measured by competitive ITC where the protein is premixed with a weak ligand (**40**).[1] Competitive measurements were performed by titrating **2**, **8** or **33** (300 µM) into the cell containing RbAp48 (30 µM) and **40** (416 µM). Other conditions were the same as for the direct measurement described above.

Ki was calculated according [1] using the following formula:

$$K_L^{app} = \frac{K_{a,L}}{1 + K_{a,X}[X]}$$

Circular dichroism

Peptides were dissolved at a concentration of 50-100 µM in a buffer containing 10 mM NaH_2PO_4 at pH 7.5. CD spectra were recorded using a Jasco J-815 CD spectrometer using a wavelength range from 190 – 260 nm and spectra were averaged over 5 measurements. Peptides were measured at 25°C at a scanning speed of 50 nm/min and a bandwidth of 1 nm.

Stability assay

Peptide stability was tested in whole cell lysate prepared from MDA-MB-231 cells using the freeze-thaw method. Peptides were dissolved in lysate (normalized to 5mg/ml protein using PBS) at 600 µM and incubated at 37°C. Samples were taken at different time points and mixed with an equal amount of MeOH containing 0.05 mg/ml ethylparaben as an internal standard. The samples were mixed and kept on ice for 15 minutes before centrifugation at 14000 rpm at 4°C for 10 min. The supernatant was carefully removed and analyzed by HPLC. Peptide peaks were integrated and surface areas compared to blank samples which did not contain lysate. Measurements were performed in duplicate.

Pull-down experiments

Nuclear lysate of MDA-MB-231 cells was prepared using a Subcellular Protein Fractionation Kit (Thermo Scientific) according to the manufacturer's instructions. DBCO agarose beads (100 µl) were washed with buffer A (50 mM Tris-HCl, 150 mM NaCl, 1 mM DTT, 1% (v/v) Triton X-100, pH 7.4, cOmplete™ EDTA-free Protease Inhibitor Cocktail) three times. The beads were incubated overnight at 4°C in a solution containing either **47**, **48** or no probe (500 µl containing 60 µM probe). After immobilization, the beads were washed again with buffer A three times followed by incubation with the nuclear lysate (500 µl) and incubated for 1.5 h at 4°C. After incubation beads were washed with buffer B (50 mM Tris-HCl, 500 mM NaCl, 1 mM DTT, 1% (v/v) Triton X-100, pH 7.4, cOmplete™ EDTA-free Protease Inhibitor Cocktail) three times. Protein was eluted by adding SDS sample buffer and loaded the mixture directly on SDS-page gel for analysis. After separation on the gel and transfer to PVDF membrane the protein was immunostained using RbAp48 primary antibody (Sigma Aldrich R3779, rabbit, 1:2000) and anti-rabbit secondary antibody (Licor IRDye® 800CW Donkey anti-Rabbit, 1:2500).

Cell painting assay

The described assay follows closely the method described by Bray et al.[2]

Initially, 5 µl U2OS medium were added to each well of a 384-well plate (PerkinElmer CellCarrier-384 Ultra). Subsequently, U2OS cell were seeded with a density of 1600 cells per well in 20 µl medium. The plate was incubated for 10 min at the ambient temperature, followed by an additional 4 h incubation (37 °C, 5 % CO_2). Compound treatment was performed with the Echo 520 acoustic dispenser (Labcyte) at final concentrations of 10 µM, 3 µM or 1 µM. Incubation with compound was performed for 20 h (37 °C, 5 % CO_2). Subsequently, mitochondria were stained with Mito Tracker Deep Red (Thermo Fisher Scientific, Cat. No. M22426). The Mito Tracker Deep Red stock solution (1 mM) was diluted to a final concentration of 100 nM in prewarmed medium. The medium was removed from the plate leaving 10 µl residual volume and 25 µl of the Mito Tracker solution were added to each well. 22 The plate was incubated for 30 min in darkness (37 °C, 5 % CO2). To fix the cells 7 µl of 18.5 % formaldehyde in PBS were added, resulting in a final formaldehyde concentration of 3.7 %. Subsequently, the plate was incubated for another 20 min in darkness (RT) and washed three times with 70 µl of PBS. (Biotek Washer Elx405). Cells were permeabilized by addition of 25 µl 0.1 % Triton X-100 to each well, followed by 15 min incubation (RT) in darkness. The cells were washed three times with PBS leaving a final volume of 10 µl. To each well 25 µl of a staining solution were added, which contains 1 % BSA, 5 µl/mL Phalloidin (Thermo Fisher Scientific, A12381), 25 µg/ml Concanavalin A (Thermo Fisher Scientific, Cat. No. C11252), 5 µg/ml Hoechst 33342 (Sigma, Cat. No. B2261-25mg), 1.5 µg/ml WGA-Alexa594 conjugate (Thermo Fisher Scientific, Cat. No. W11262) and 1.5 µM µl/ml SYTO 14 solution (Thermo Fisher Scientific, Cat. No. S7576). The plate is incubated for 30 min (RT) in darkness and washed three times with 70 µl PBS. After the final washing step the PBS was not aspirated. The plates were sealed and centrifuged for 1 min at 500 rpm.

The plates were prepared in triplicates with shifted layouts to reduce plate effects and imaged using a Micro XL High-Content Screening System (Molecular Devices) in 5 channels (DAPI: Ex350-400/ Em410-480; FITC: Ex470-500/ Em510-540; Spectrum Gold: Ex520-545/ Em560- 585; TxRed: Ex535-585/ Em600-650; Cy5: Ex605-650/ Em670-715) with 9 sites per well and 20x magnification (binning 2).

The generated images were processed with the CellProfiler package (https://cellprofiler.org/, version 3.0.0) on a computing cluster of the Max Planck Society to extract 1716 cell features (parameters) per microscope site. The data was then further aggregated as medians per well (9 sites -> 1 well), then over the three replicates.

Further analysis was performed with custom Python (https://www.python.org/) scripts using the Pandas (https://pandas.pydata.org/) and Dask (https://dask.org/) data processing libraries (separate publication to follow).

From the total set of 1716 parameters a subset of highly reproducible and robust parameters was determined using the procedure described by Woehrmann et al.[3] in the following way: Two biological repeats of one plate containing reference compounds were analysed. For every parameter, its full profile over each whole plate was calculated. If the profiles from the two repeats showed a similarity >= 0.8 (see below), the parameter was added to the set. This procedure was only performed once and resulted in a set of 579 robust parameters out of the total of 1716 that was used for all further analyses.

To determine the phenotypic fingerprint for each test compound Z-scores were then calculated for each parameter as how many times the Median Absolute Deviation (MAD) of the controls the measured parameter value of a test compound deviates from the Median of the controls:

$$z - score = \frac{value_{meas.} - \ Median_{controls}}{MAD_{controls}}$$

The morphological compound fingerprint is then the list of z-scores of all parameters for one compound.

In addition to the morphological fingerprint, an induction value was determined for each compound as the fraction of significantly changed parameters, in percent:

$$Induction\ [\%] = \frac{number\ of\ parameter\ with\ abs.values > 3}{total\ number\ of\ parameters}$$

Similarities of morphological fingerprints were calculated from the correlation distances between two fingerprints (https://docs.scipy.org/doc/scipy/reference/generated/scipy.spatial.distance.correlation.html; Similarity = 1 - Correlation Distance) and the compounds with the most similar fingerprints were determined from a set of 3000 reference compounds that was also measured in the assay.

p53 expression level assay

U2OS cells were incubated with 10 µM compound **49**, **50** or DMSO (DMSO 0.5%) for 24 h followed by lysis for western blotting analysis. p53 expression was monitored using fluorescent antibody. Signal ration between p53 and loading control (alpha tubulin) was measured. Data are mean value of two biological replicates.

[1] A. Velazquez-Campoy, E. Freire, *Nat. Protoc.* **2006**, *1*, 186–191.

[2] M.-A. Bray, S. Singh, H. Han, C. T. Davis, B. Borgeson, C. Hartland, M. Kost-Alimova, S. M. Gustafsdottir, C. C. Gibson, A. E. Carpenter, *Nat. Protoc.* **2016**, *11*, 1757–1774.

[3] M. H. Woehrmann, W. M. Bray, J. K. Durbin, S. C. Nisam, A. K. Michael, E. Glassey, J. M. Stuart, R. S. Lokey, *Mol. Biosyst.* **2013**, *9*, 2604.

[4] J. Lu, S. Guan, Y. Zhao, Y. Yu, Y. Wang, Y. Shi, X. Mao, K. L. Yang, W. Sun, X. Xu, et al., *Oncotarget* **2016**, *7*, 82757–82769.

[5] A. P. Taylor, M. Swewczyk, S. Kennedy, V. V. Trush, H. Wu, H. Zeng, A. Dong, R. Ferreira De Freitas, J. Tatlock, R. A. Kumpf, et al., *J. Med. Chem.* **2019**, *62*, 7669–7683.

[6] J.-D. Fan, P.-J. Lei, J.-Y. Zheng, X. Wang, S. Li, H. Liu, Y.-L. He, Z.-N. Wang, G. Wei, X. Zhang, et al., *PLoS One* **2015**, *10*, e0116782.

[7] L. J. B. Malhab, S. Descamps, B. Delaval, D. P. Xirodimas, *Sci. Rep.* **2016**, *6*, 37775.

[8] Y. Zhu, L. Xu, J. Zhang, X. Hu, Y. Liu, H. Yin, T. Lv, H. Zhang, L. Liu, H. An, et al., *Cancer Sci.* **2013**, *104*, 1052–1061.

[9] R.-J. Chen, W.-S. Lee, Y.-C. Liang, J.-K. Lin, Y.-J. Wang, C.-H. Lin, J.-Y. Hsieh, C.-C. Chaing, Y.-S. Ho, *Toxicol. Appl. Pharmacol.* **2000**, *169*, 132–141.

Overview of compounds

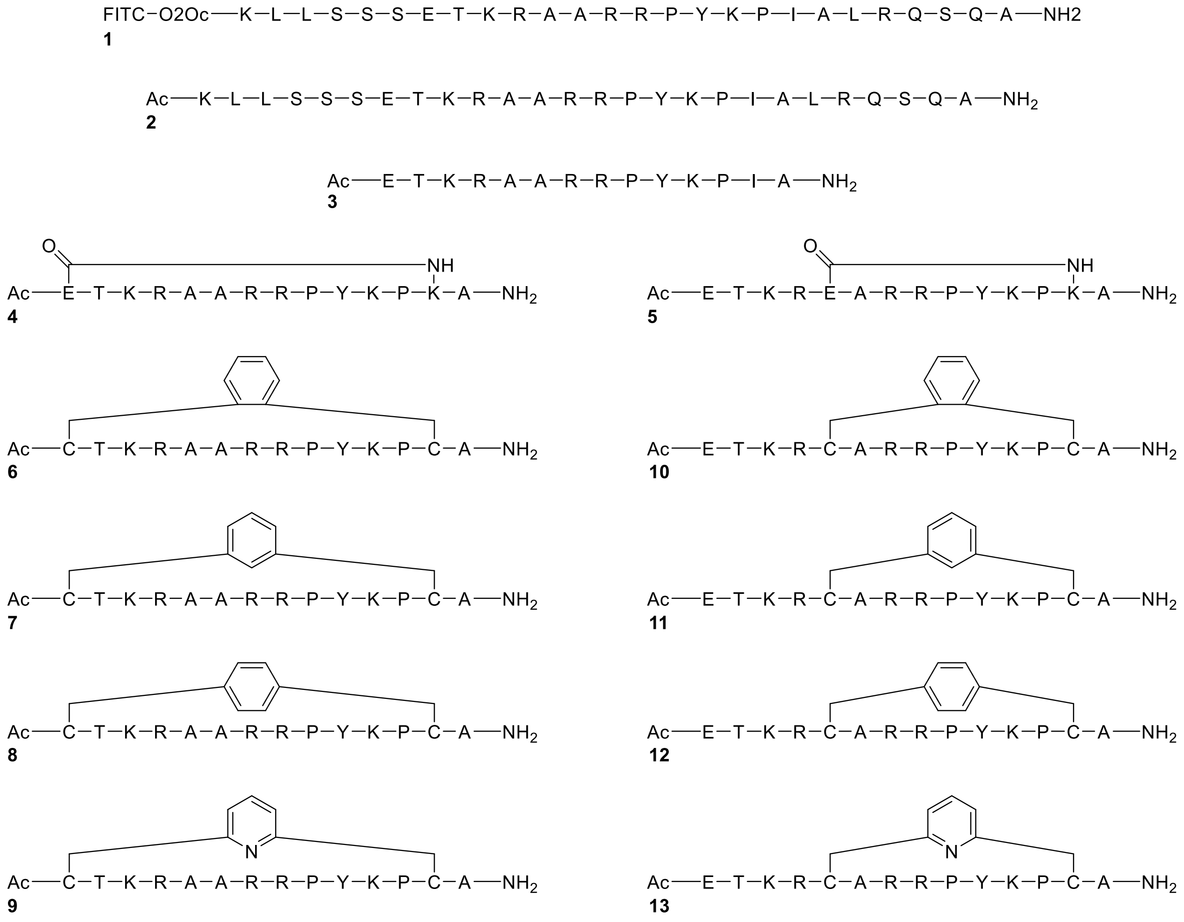

Supplemental figure 1: Peptides **1 – 13**

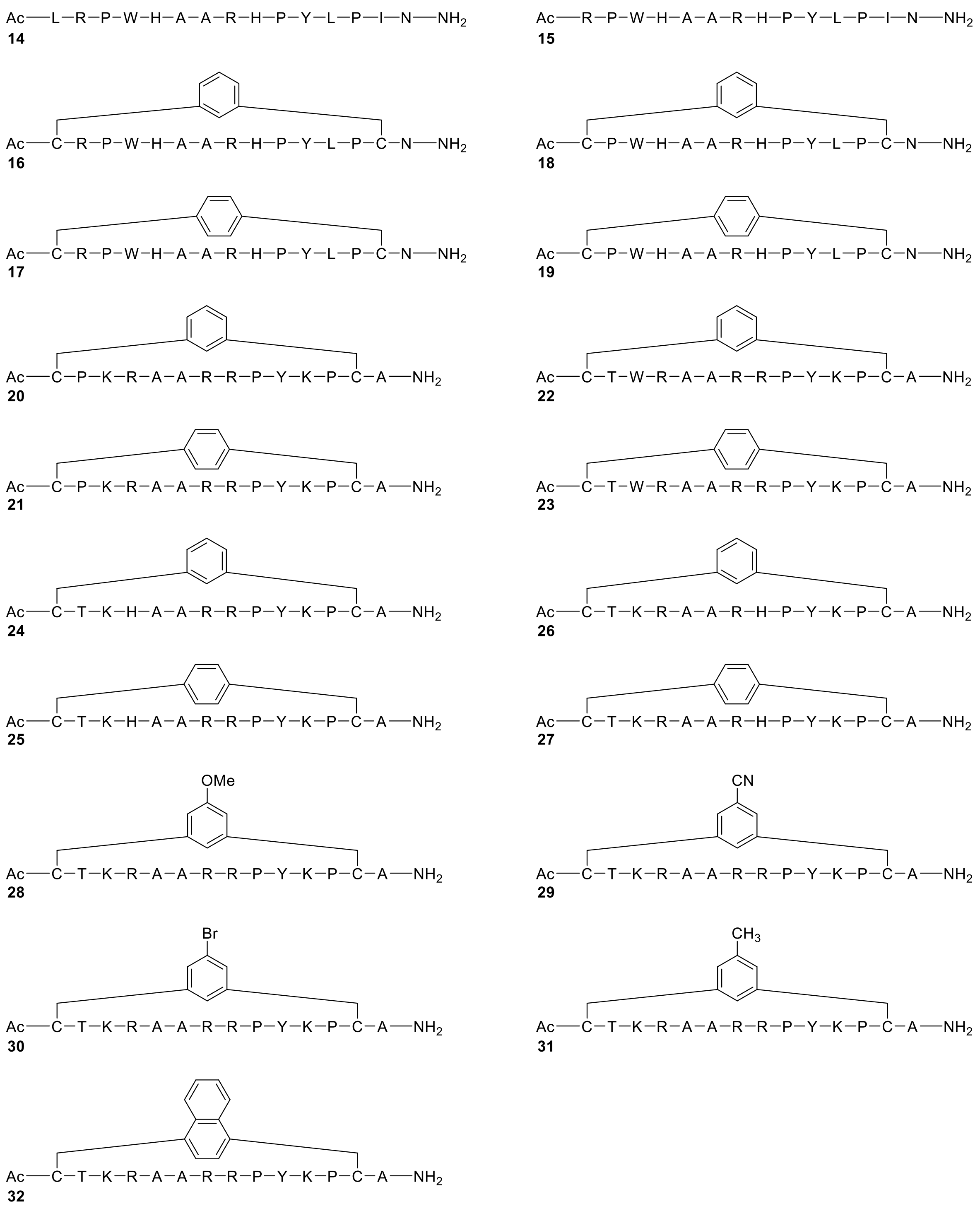

Supplemental figure 2: Peptides **14** – **32**

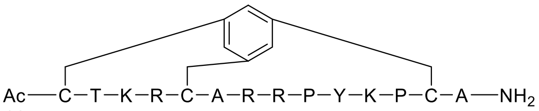

33

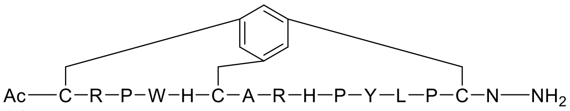

34

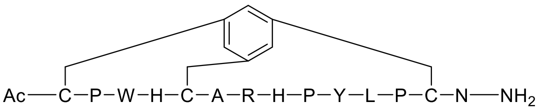

35

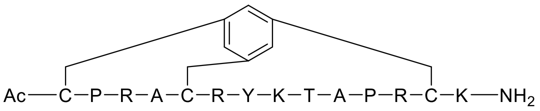

36

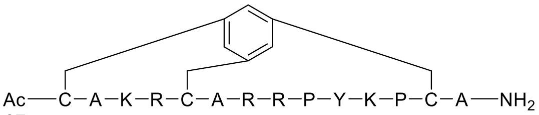

37

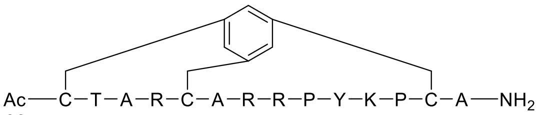

38

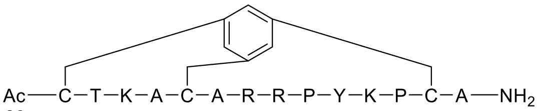

39

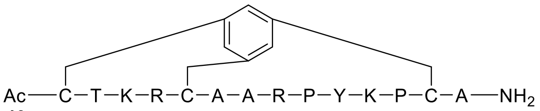

40

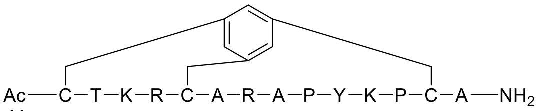

41

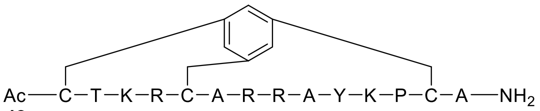

42

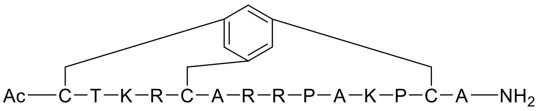

43

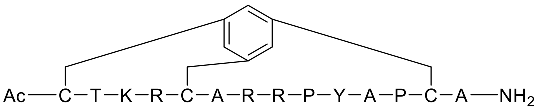

44

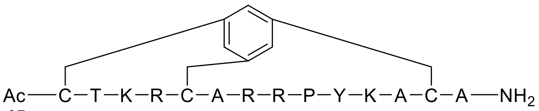

45

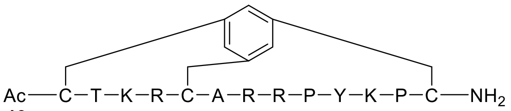

46

Supplemental figure 3: Peptides **33** – **46**

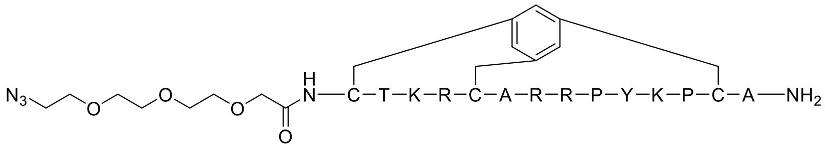

47

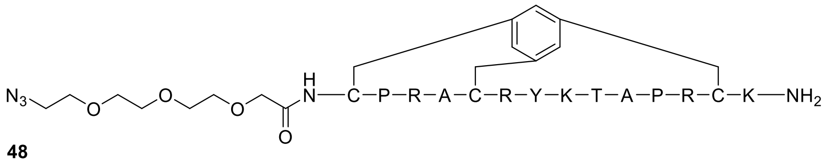

48

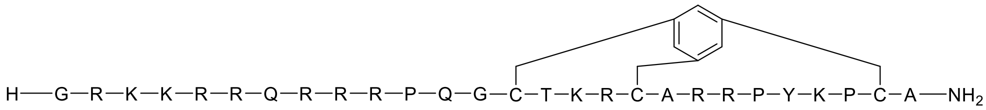

49

H—G—R—K—K—R—R—Q—R—R—R—P—Q—NH_2

50

Supplemental figure 4: Peptides **47** – **50**

Fluorescence polarization results

Supplemental Table 2: Sequence, cyclization strategy and IC_{50} values for all peptides.

Peptide[a]	Sequence/mutation[b]	Cyclization	IC_{50} (nM)
	676 680 688		
**1**	FITC-PEG-KLLSSS**E**TKR**A**ARRPYKP**I**ALRQSQA-NH_2		-
**2**	Ac-KLLSSSETKRAARRPYKPIALRQSQA-NH_2		13.4 ± 3.0
**3**	Ac-ETKRAARRPYKPIA-NH_2		2621 ± 786
**4**	Ac-ETKRAARRPYKPKA-NH_2	amide	47.7 ± 12.5
**5**	Ac-ETKREARRPYKPKA-NH_2	amide	125.6 ± 32.3
**6**	Ac-CTKRAARRPYKPCA-NH_2	ortho xylene	77.4 ± 6.5
**7**	Ac-CTKRAARRPYKPCA-NH_2	meta xylene	18.7 ± 4.1
**8**	Ac-CTKRAARRPYKPCA-NH_2	para xylene	15.1 ± 5.4
**9**	Ac-CTKRAARRPYKPCA-NH_2	meta pyridine	34.9 ± 14.0
**10**	Ac-ETKRCARRPYKPCA-NH_2	ortho xylene	321.7 ± 56.8
**11**	Ac-ETKRCARRPYKPCA-NH_2	meta xylene	388.0 ± 69.8
**12**	Ac-ETKRCARRPYKPCA-NH_2	para xylene	2342 ± 363
**13**	Ac-ETKRCARRPYKPCA-NH_2	meta pyridine	146.8 ± 10.7
	490 495 503		
**14** (R1)	Ac-**L**RPWH**A**ARHPYLP**I**N-NH_2	-	>10000
**15** (R1)	Ac-RPWHAARHPYLPIN-NH_2	-	>10000
**16** (R1)	Ac-CRPWHAARHPYLPCN-NH_2	meta xylene	>10000
**17** (R1)	Ac-CRPWHAARHPYLPCN-NH_2	para xylene	>10000
**18** (R1)	Ac-CPWHAARHPYLPCN-NH_2	meta xylene	>10000
**19** (R1)	Ac-CPWHAARHPYLPCN-NH_2	para xylene	>10000
**20**	Ac-C**P**KRAARRPYKPCA-NH_2	meta xylene	22.3 ± 0.03
**21**	Ac-C**P**KRAARRPYKPCA-NH_2	para xylene	25.0 ± 7.3
**22**	Ac-CT**W**RAARRPYKPCA-NH_2	meta xylene	315.5 ± 57.8
**23**	Ac-CT**W**RAARRPYKPCA-NH_2	para xylene	245.0 ± 29.3

**24**	Ac-CTKHAARRPYKPCA-NH_2	meta xylene	265.1 ± 7.4
**25**	Ac-CTKHAARRPYKPCA-NH_2	para xylene	303.0 ± 85.9
**26**	Ac-CTKRAARHPYKPCA-NH_2	meta xylene	512.4 ± 48.4
**27**	Ac-CTKRAARHPYKPCA-NH_2	para xylene	335.2 ± 65.0
**28**	Ac-CTKRAARRPYKPCA-NH_2	5-methoxyxylene	38.4 ± 3.8
**29**	Ac-CTKRAARRPYKPCA-NH_2	5-cyanoxylene	43.8 ± 4.7
**30**	Ac-CTKRAARRPYKPCA-NH_2	5-bromoxylene	31.0 ± 3.7
**31**	Ac-CTKRAARRPYKPCA-NH_2	5-methylxylene	40.4 ± 1.5
**32**	Ac-CTKRAARRPYKPCA-NH_2	1,4-naphtalene	43.7 ± 6.4
**33**	Ac-CTKRCARRPYKPCA-NH_2	mesitylene	12.3 ± 2.0
**34**	Ac-CRPWHCARHPYLPCN-NH_2	mesitylene	152.9 ± 2.4
**35**	Ac-CPWHCARHPYLPCN-NH_2	mesitylene	7485 ± 3024
**36** (R2 scrambled)	Ac-CPRACRYKTAPRCK-NH_2	mesitylene	>10000
**37**	Ac-CAKRCARRPYKPCA-NH_2	mesitylene	12.4 ± 3.2
**38**	Ac-CTARCARRPYKPCA-NH_2	mesitylene	262.9 ± 42.7
**39**	Ac-CTKACARRPYKPCA-NH_2	mesitylene	77.4 ± 9.6
**40**	Ac-CTKRCAARPYKPCA-NH_2	mesitylene	4319 ± 974
**41**	Ac-CTKRCARAPYKPCA-NH_2	mesitylene	24.6 ± 3.9
**42**	Ac-CTKRCARRAYKPCA-NH_2	mesitylene	131.3 ± 15.3
**43**	Ac-CTKRCARRPAKPCA-NH_2	mesitylene	38.3 ± 1.9
**44**	Ac-CTKRCARRPYAPCA-NH_2	mesitylene	19.8 ± 3.6
**45**	Ac-CTKRCARRPYKACA-NH_2	mesitylene	15.6 ± 2.2
**46**	Ac-CTKRCARRPYKPC-NH_2	mesitylene	20.5 ± 1.4
**47**	N_3-PEG-CTKRCARRPYKPCA-NH_2	mesitylene	13.9 ± 5.6
**48** (R2 scrambled)	N_3-PEG-CPRACRYKTAPRCK-NH_2	mesitylene	>10000
**49** (Tat-modified)	H-GRKKRRQRRRPQGCTKRCARRPYKPCA-NH_2	mesitylene	4.4 ± 1.4
**50** (Tat)	H-GRKKRRQRRRPQ-NH_2	-	1239 ± 358

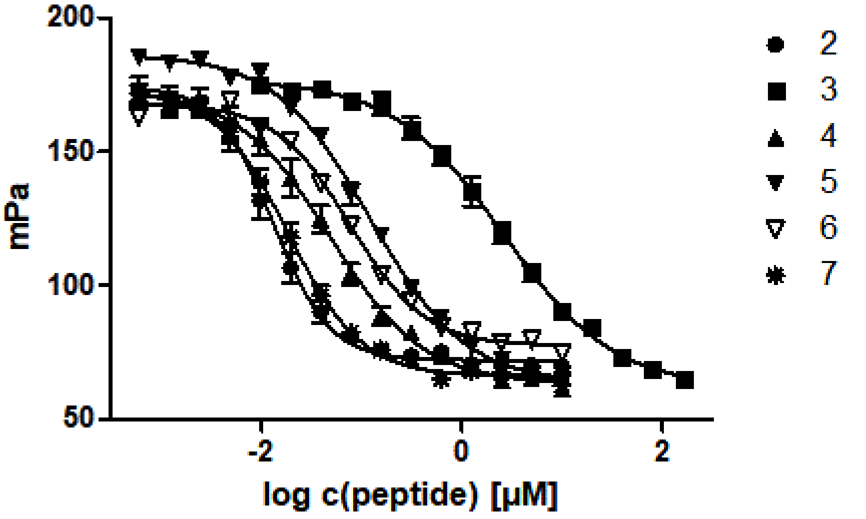

Supplemental figure 5: Competitive fluorescence polarization binding curves for peptides **2**-**7**

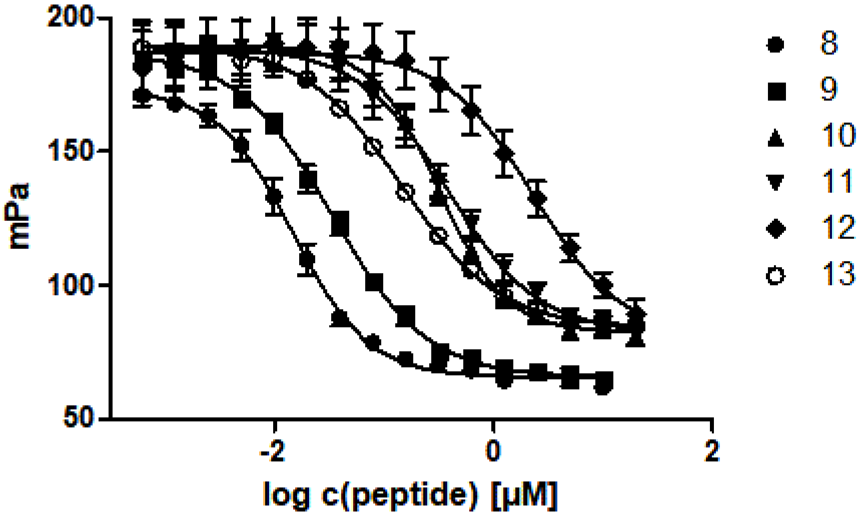

Supplemental figure 6: Competitive fluorescence polarization binding curves for peptides **8**-**13**

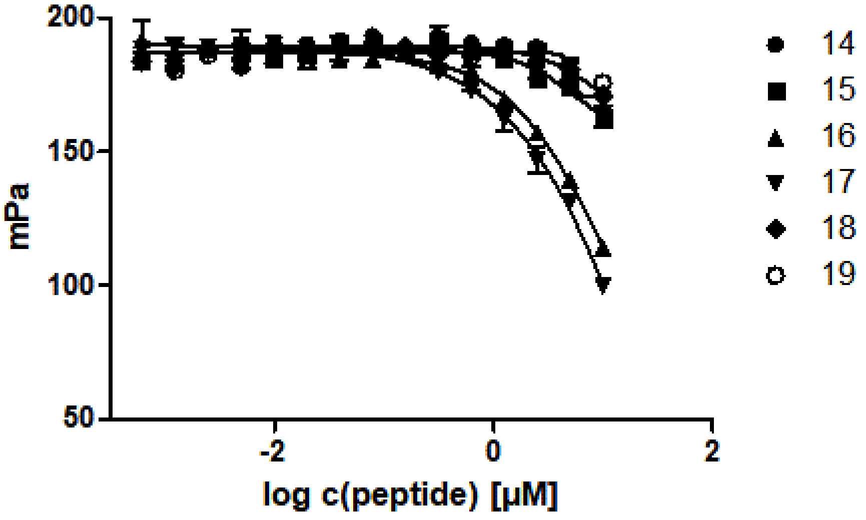

Supplemental figure 7: Competitive fluorescence polarization binding curves for peptides **14**-**19**

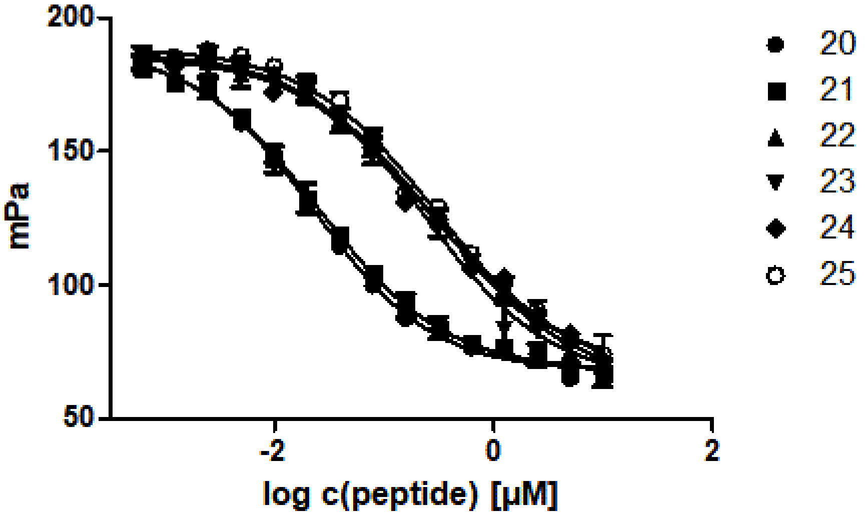

Supplemental figure 8: Competitive fluorescence polarization binding curves for peptides **20**-**25**

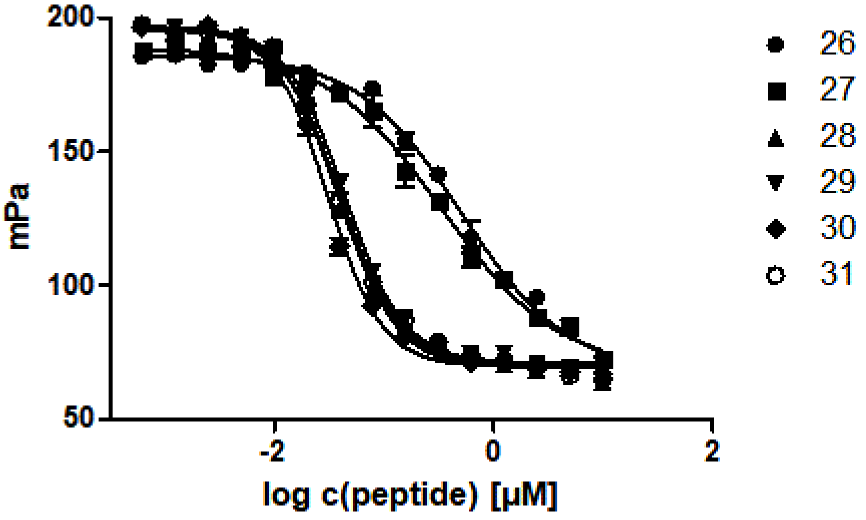

Supplemental figure 9: Competitive fluorescence polarization binding curves for peptides **26**-**31**

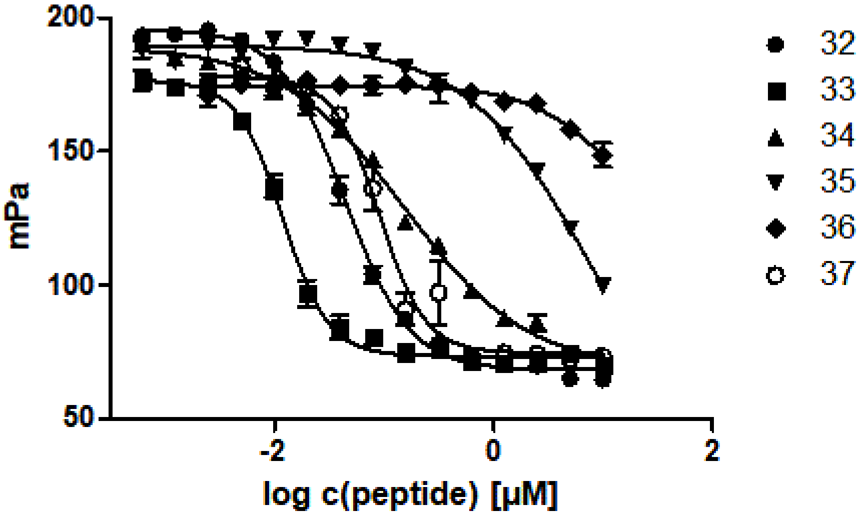

Supplemental figure 10: Competitive fluorescence polarization binding curves for peptides **32**-**37**

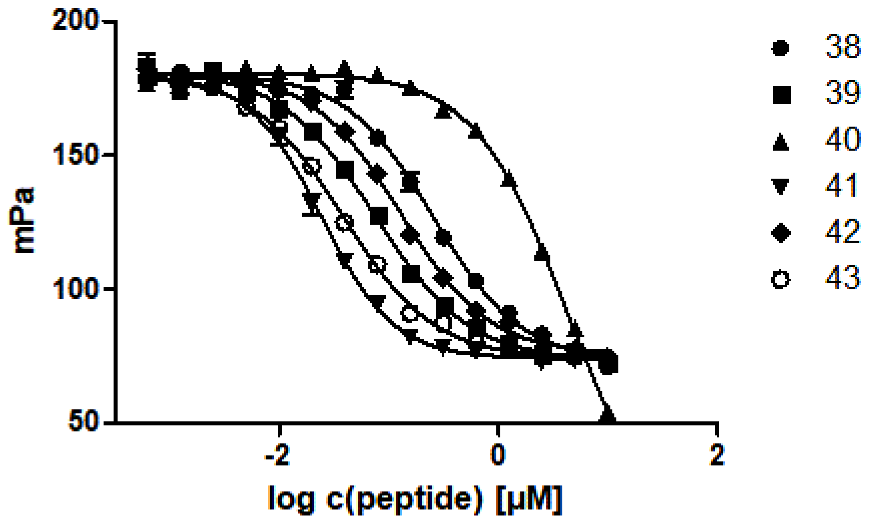

Supplemental figure 11: Competitive fluorescence polarization binding curves for peptides **38**-**43**

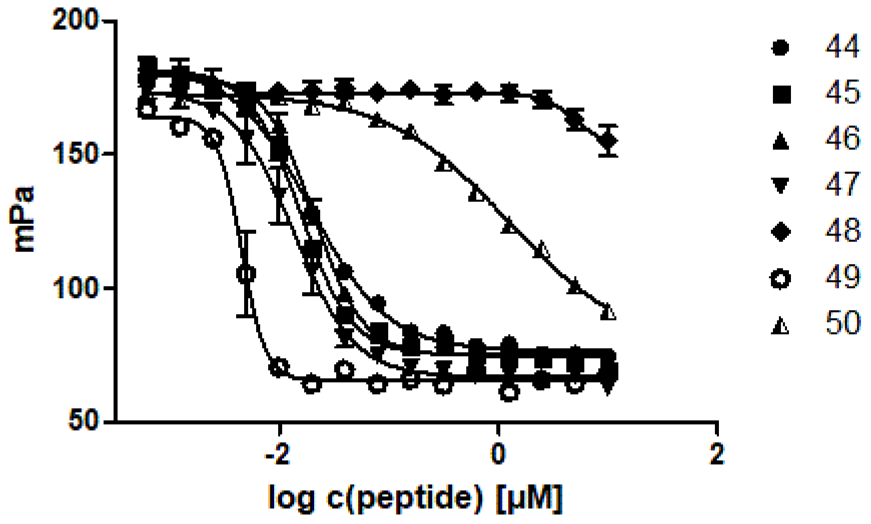

Supplemental figure 12: Competitive fluorescence polarization binding curves for peptides **44**-**50**

ITC thermograms

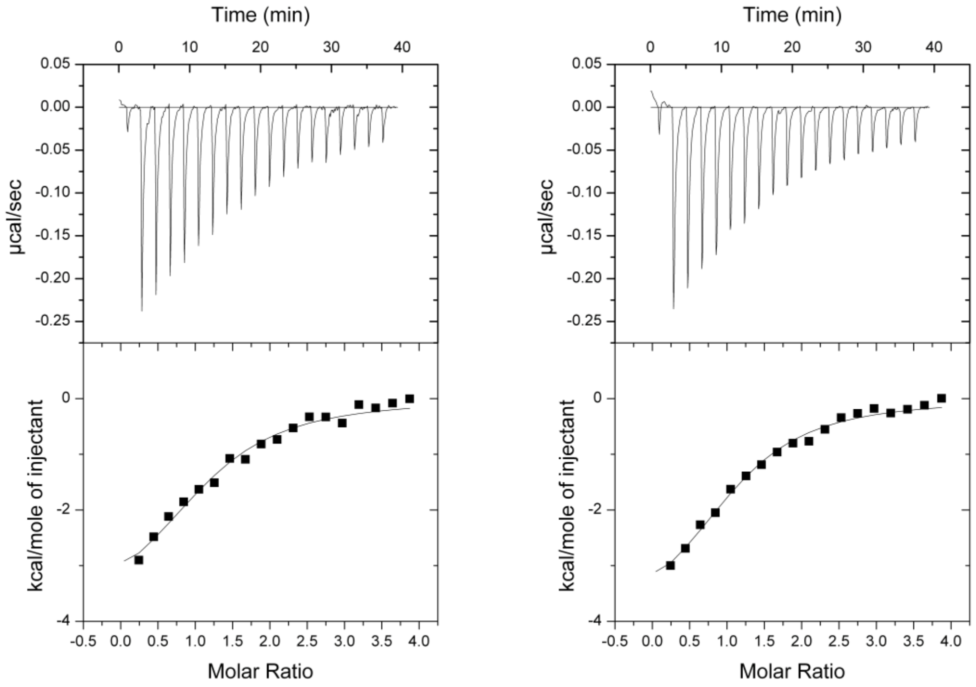

Supplemental figure 13: Direct titration of **40** to RbAp48 (duplicate)

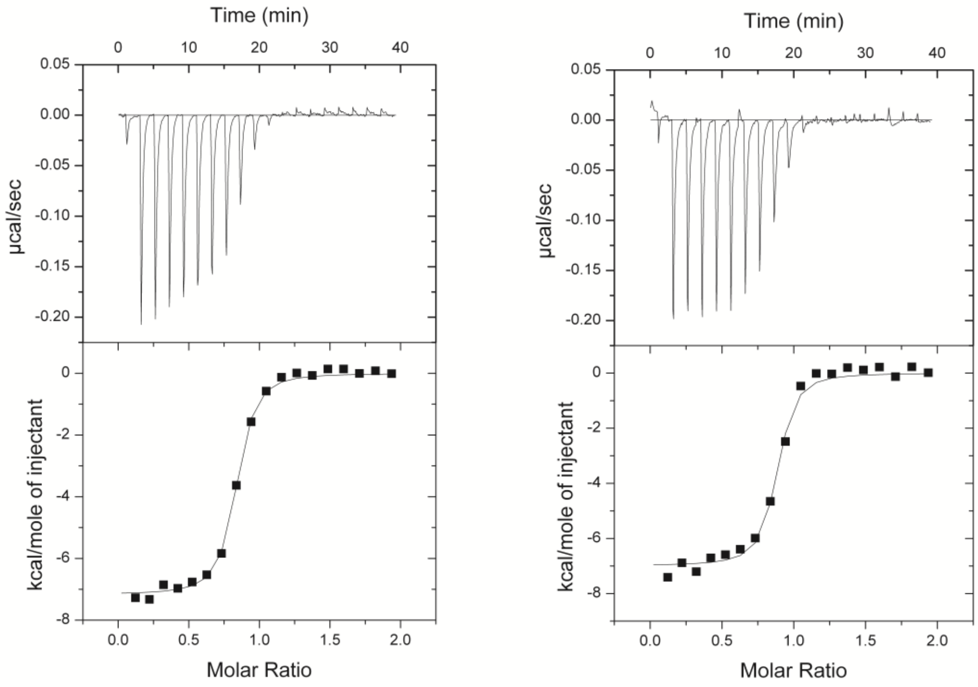

Supplemental figure 14: Competitive titration of **2** to RbAp48 premixed with **40** (duplicate)

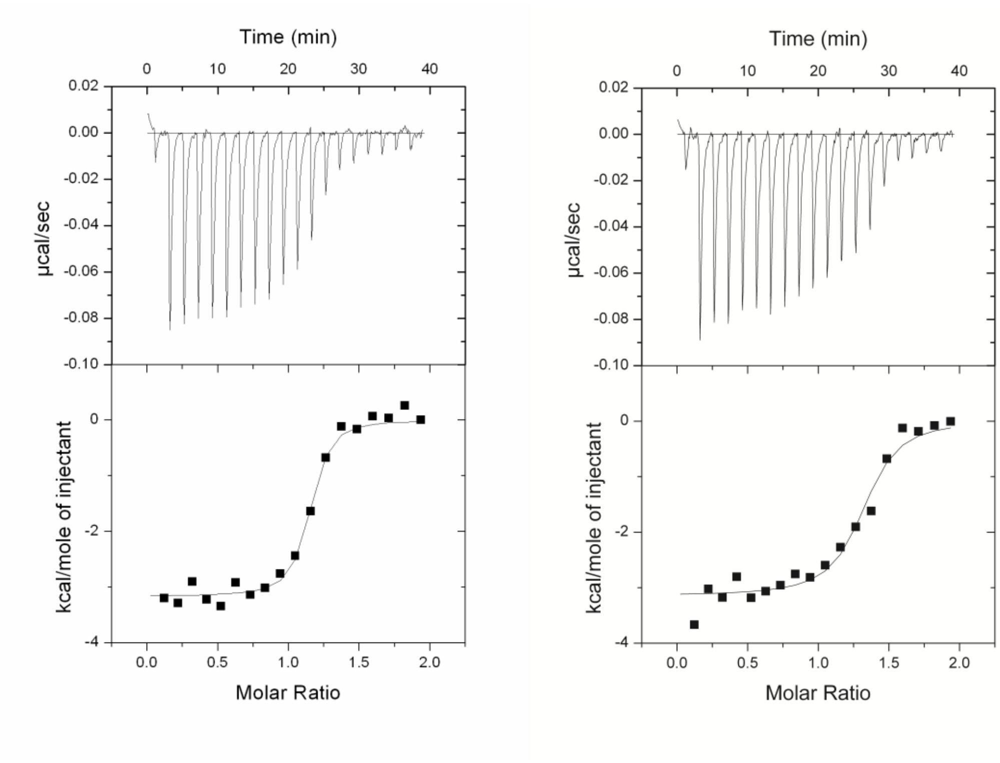

Supplemental figure 15: Competitive titration of **33** to RbAp48 premixed with **40** (duplicate)

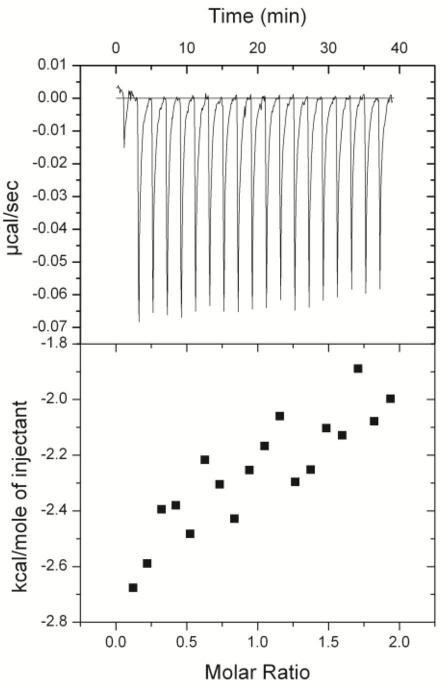

Supplemental figure 16: Competitive titration of **8** to RbAp48 premixed with **40**

Circular dichroism spectra

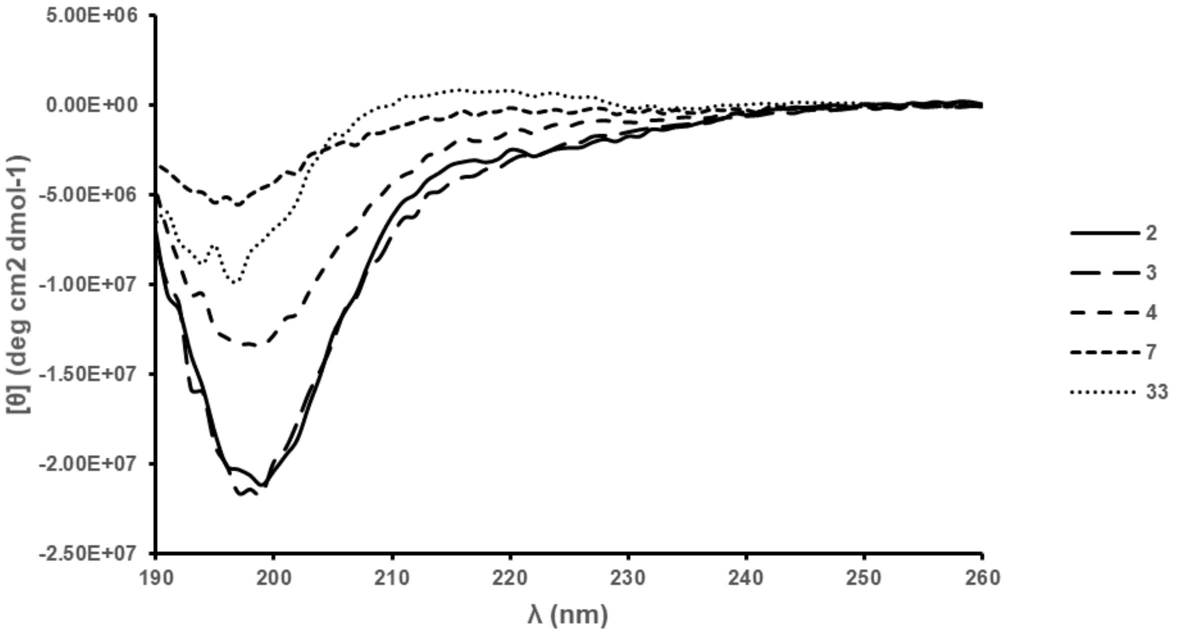

Supplemental figure 17: Circular dichroism spectra of compounds **2**, **3**, **4**, **7**, and **33**. All spectra indicate a random coil conformation of the peptides in solution.

Peptide stability in cell lysate

Supplemental table 3: Stability analysis of compound **3**

T (min)	% left
0	100
5	67.5
15	49.2
60	0

Supplemental table 4: Stability analysis of compound **8**

T (min)	% left
0	100
5	67.8
15	41.0
60	0

Supplemental table 5: Stability analysis of compound **33** in cell lysate

T (min)	% left
0	100
60	57.8
120	41.4
240	0

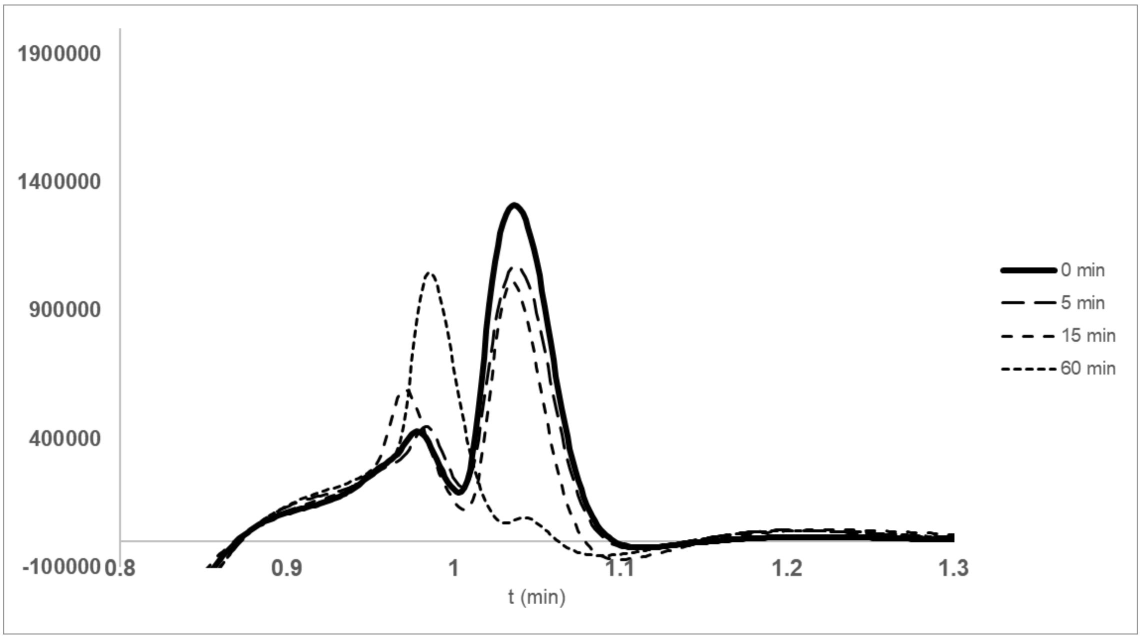

Supplemental figure 18: Degradation of **3** analyzed by HPLC

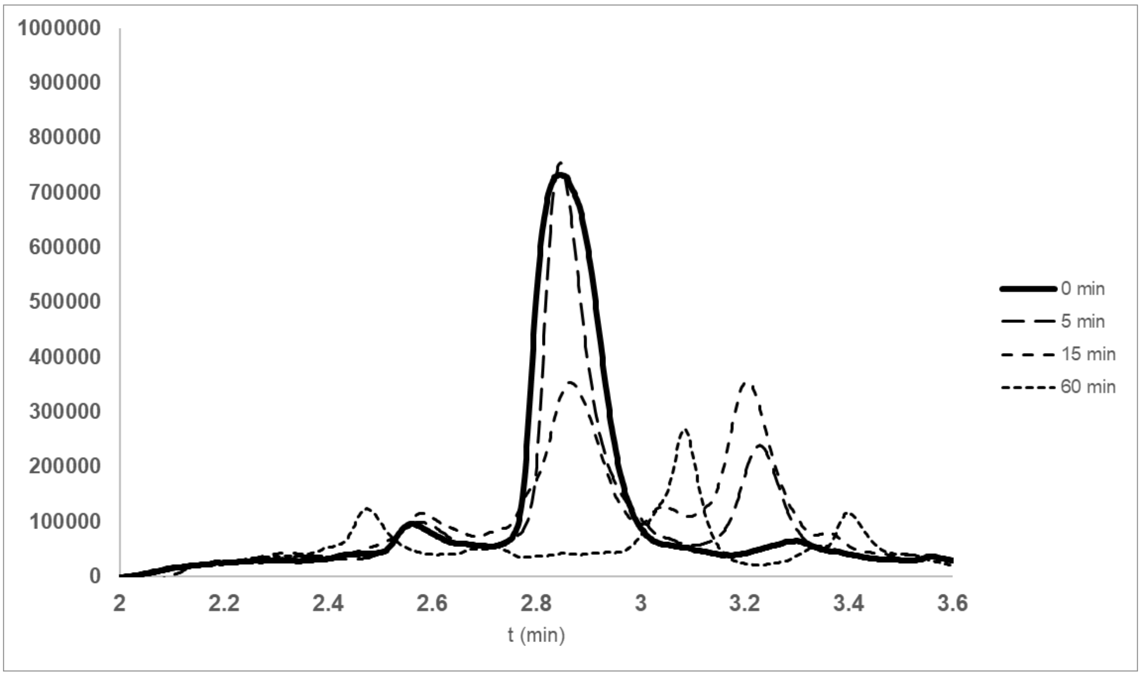

Supplemental figure 19: Degradation of **8** analyzed by HPLC

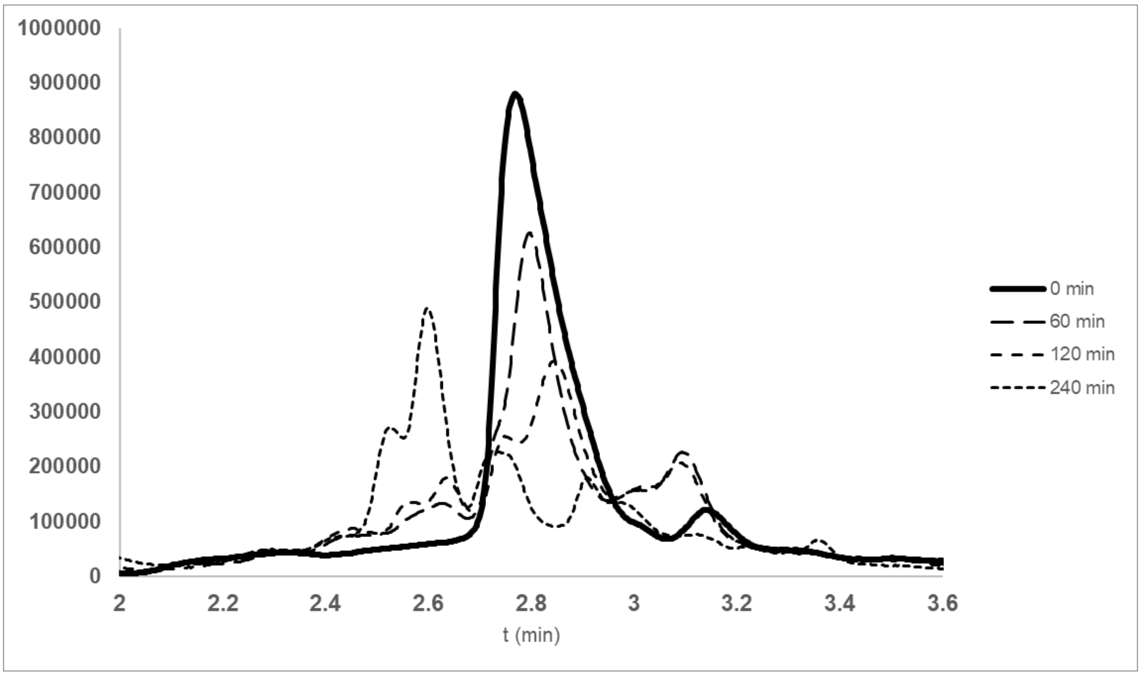

Supplemental figure 20: Degradation of **33** analyzed by HPLC

Supplemental table 6: Reference compounds with similar cell painting profiles as compound 49 for which direct p53 enhancement has been shown.

Compound	Structure	Biosimilarity (%)	References
MI-773	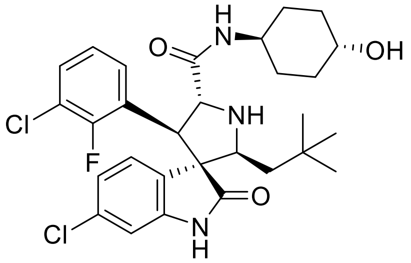	73.0	[4]
PFI-5	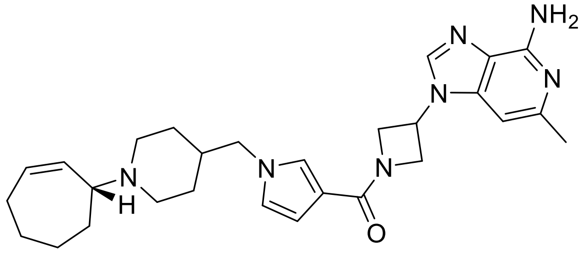	65.1	[5]
BIX01294	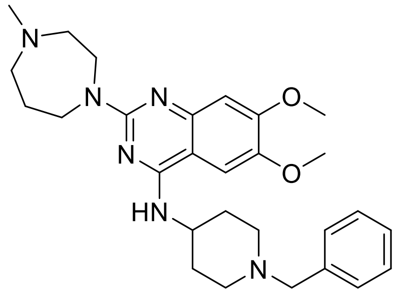	64.9	[6]
Pevonedistat	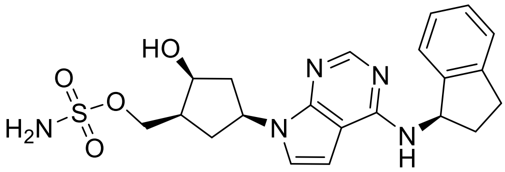	63.6	[7]
Sunitinib	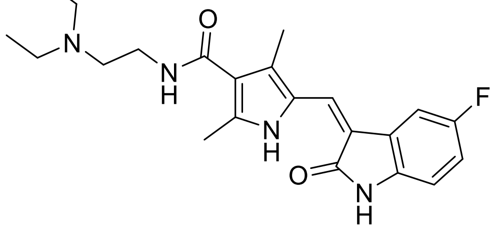	63.5	[8]
Ketoconazole	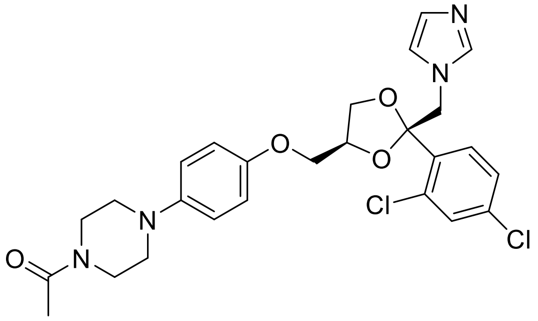	61.9	[9]

References

[1] A. Velazquez-Campoy, E. Freire, *Nat. Protoc.* **2006**, *1*, 186–191.

[2] M.-A. Bray, S. Singh, H. Han, C. T. Davis, B. Borgeson, C. Hartland, M. Kost-Alimova, S. M. Gustafsdottir, C. C. Gibson, A. E. Carpenter, *Nat. Protoc.* **2016**, *11*, 1757–1774.

[3] M. H. Woehrmann, W. M. Bray, J. K. Durbin, S. C. Nisam, A. K. Michael, E. Glassey, J. M. Stuart, R. S. Lokey, *Mol. Biosyst.* **2013**, *9*, 2604.

[4] J. Lu, S. Guan, Y. Zhao, Y. Yu, Y. Wang, Y. Shi, X. Mao, K. L. Yang, W. Sun, X. Xu, et al., *Oncotarget* **2016**, *7*, 82757–82769.

[5] A. P. Taylor, M. Swewczyk, S. Kennedy, V. V. Trush, H. Wu, H. Zeng, A. Dong, R. Ferreira De Freitas, J. Tatlock, R. A. Kumpf, et al., *J. Med. Chem.* **2019**, *62*, 7669–7683.

[6] J.-D. Fan, P.-J. Lei, J.-Y. Zheng, X. Wang, S. Li, H. Liu, Y.-L. He, Z.-N. Wang, G. Wei, X. Zhang, et al., *PLoS One* **2015**, *10*, e0116782.

[7] L. J. B. Malhab, S. Descamps, B. Delaval, D. P. Xirodimas, *Sci. Rep.* **2016**, *6*, 37775.

[8] Y. Zhu, L. Xu, J. Zhang, X. Hu, Y. Liu, H. Yin, T. Lv, H. Zhang, L. Liu, H. An, et al., *Cancer Sci.* **2013**, *104*, 1052–1061.

[9] R.-J. Chen, W.-S. Lee, Y.-C. Liang, J.-K. Lin, Y.-J. Wang, C.-H. Lin, J.-Y. Hsieh, C.-C. Chaing, Y.-S. Ho, *Toxicol. Appl. Pharmacol.* **2000**, *169*, 132–141.

HPLC Analysis of compounds **1** – **50**

Analytical method A:

Peptides were analyzed on a Macherey Nagel, NUCLEODUR C18 column, 3 µm, 50 x 2.1 mm at a flow rate of 0.56 ml/min. A linear gradient of mobile phase: A = H_2O + 0.1% TFA and B = CH_3CN +0.1 % TFA The gradient started with 5 % ACN and linearly increased to 65 % over 14 min with detection at 210 nm.

Analytical method B:

Peptides were analyzed on a Waters Acquity CSH C18 column, 130 Å, 1.7 µm, 50 x 2.1 mm at a flow rate of 0.5 ml/min at 45 °C; A linear gradient of mobile phase: A = H_2O + 10 mM formic acid, 1 mM ammonia and 0.03% TFA and B = CH_3CN/H_2O 95/5 (vol/vol-%) + 10 mM formic acid, 1 mM ammonia and 0.03% TFA was used with detection at 220 nm.

Analytical method C:

Peptides were analyzed on a Waters Acquity HSS T3 C18 column, 130 Å, 1.8 µm, 50 x 2.1 mm at a flow rate of 0.4 ml/min at 45 °C. A linear gradient of mobile phase: A = H_2O + 10 mM formic acid, 1 mM ammonia and 0.03% TFA and B = CH_3CN/H_2O 95/5 (vol/vol-%) + 10 mM formic acid, 1 mM ammonia and 0.03% TFA was used with detection at 220 nm.

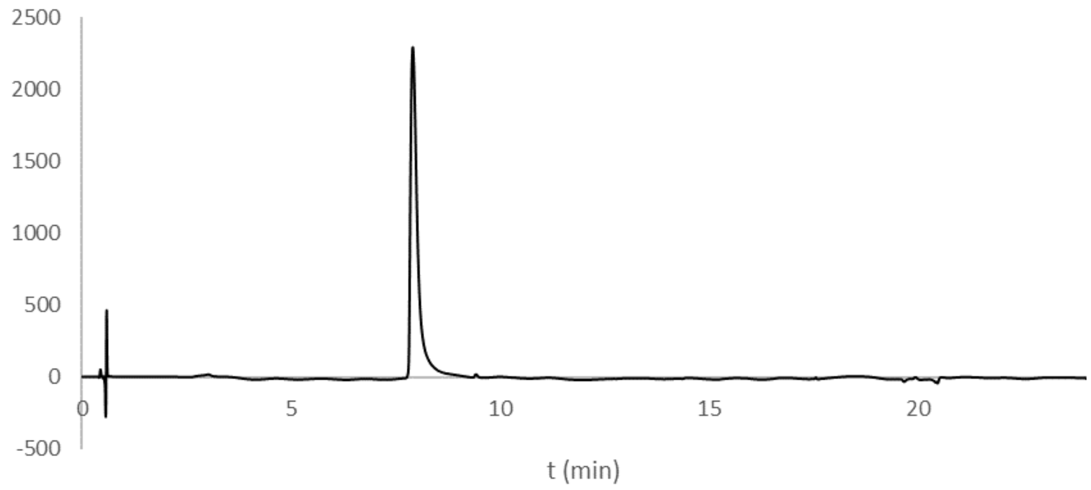

Supplemental figure 22: HPLC analysis of compound **1** (method A)

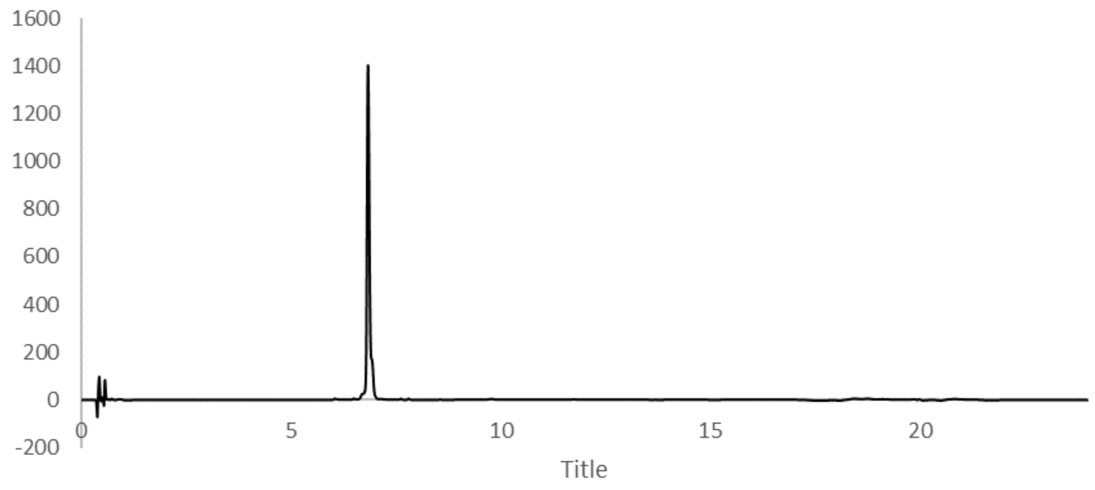

Supplemental figure 23: HPLC analysis of compound **2** (method A)

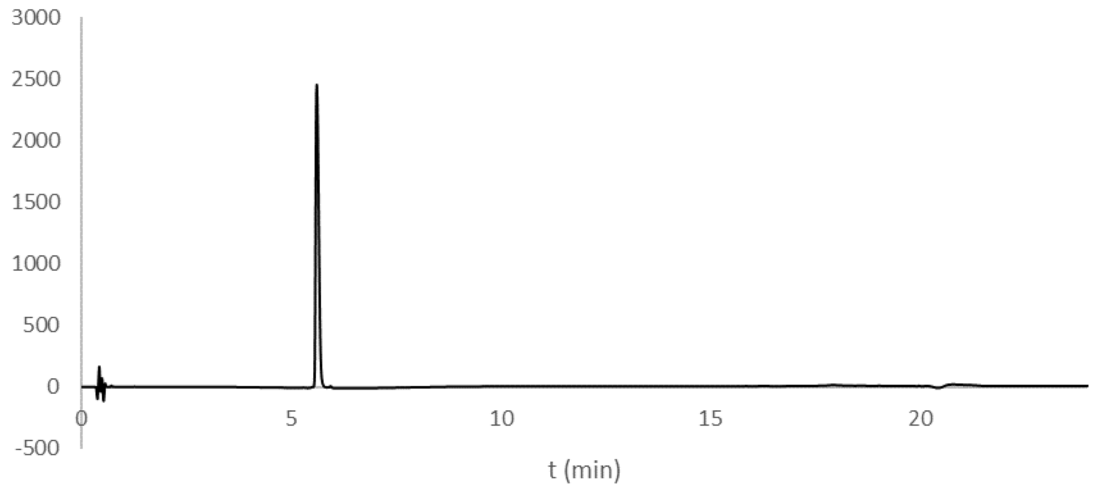

Supplemental figure 24: HPLC analysis of compound **3** (method A)

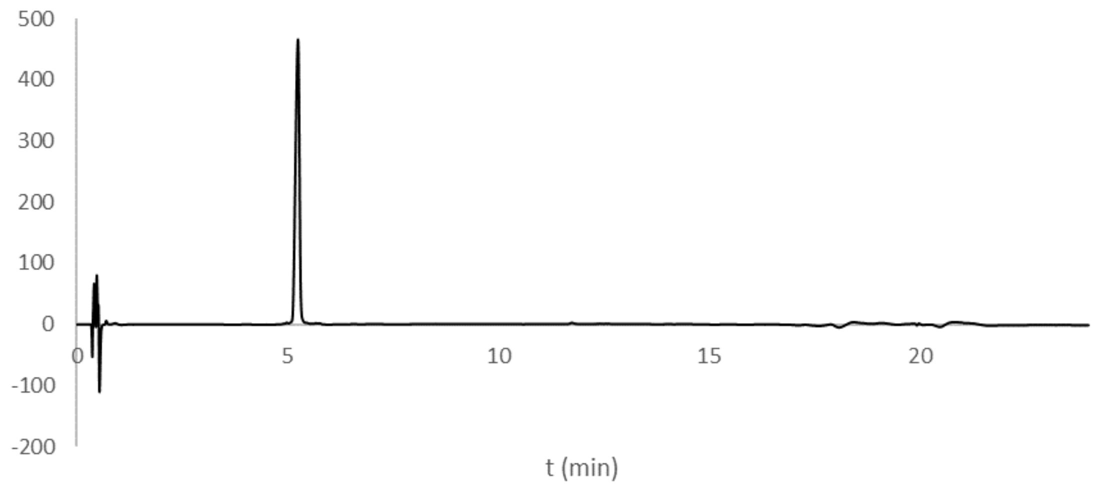

Supplemental figure 25: HPLC analysis of compound **4** (method A)

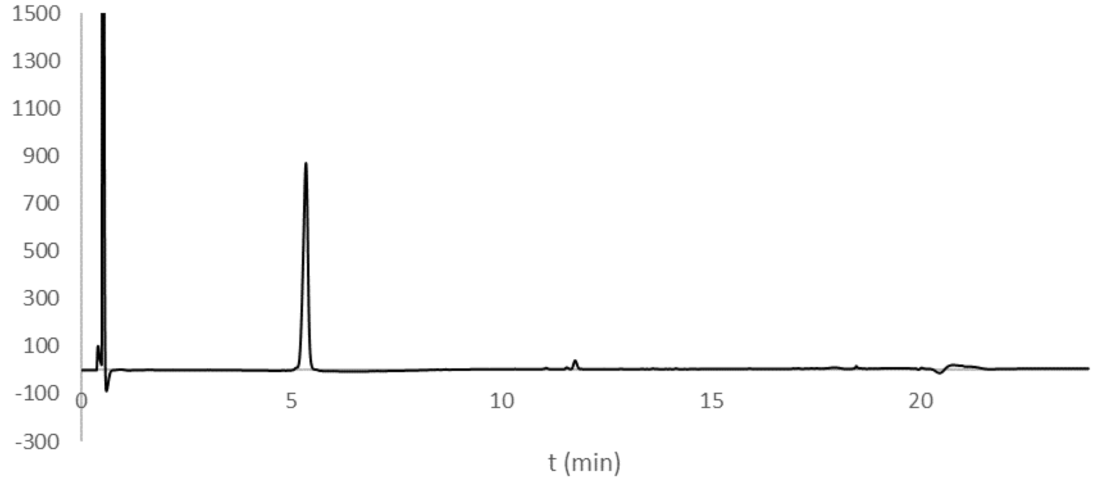

Supplemental figure 26: HPLC analysis of compound **5** (method A)

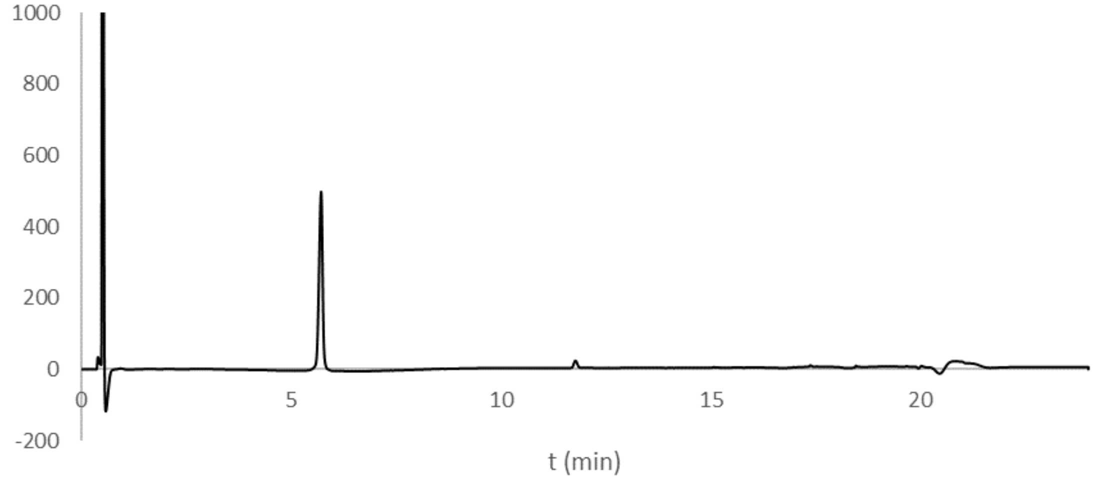

Supplemental figure 27: HPLC analysis of compound **6** (method A)

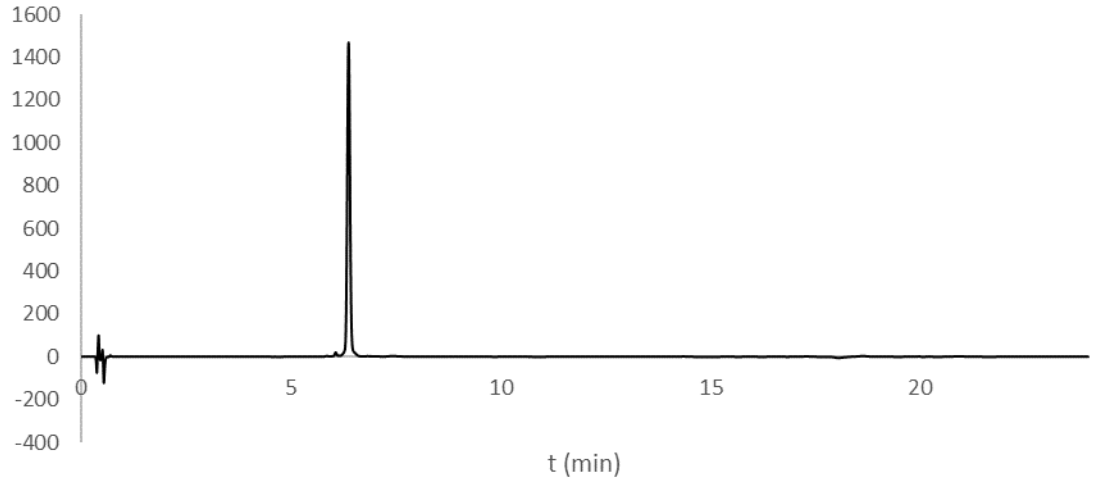

Supplemental figure 28: HPLC analysis of compound **7** (method A)

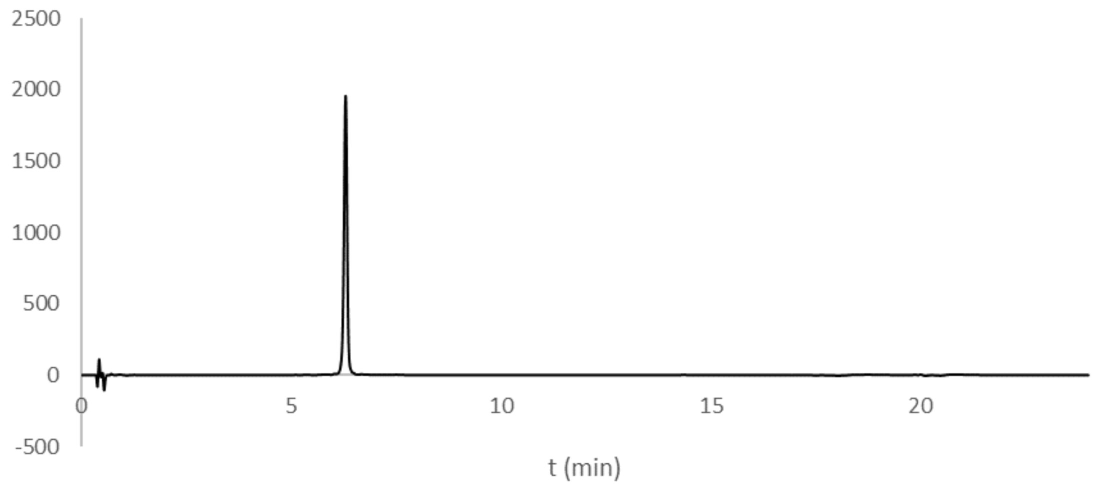

Supplemental figure 29: HPLC analysis of compound **8** (method A)

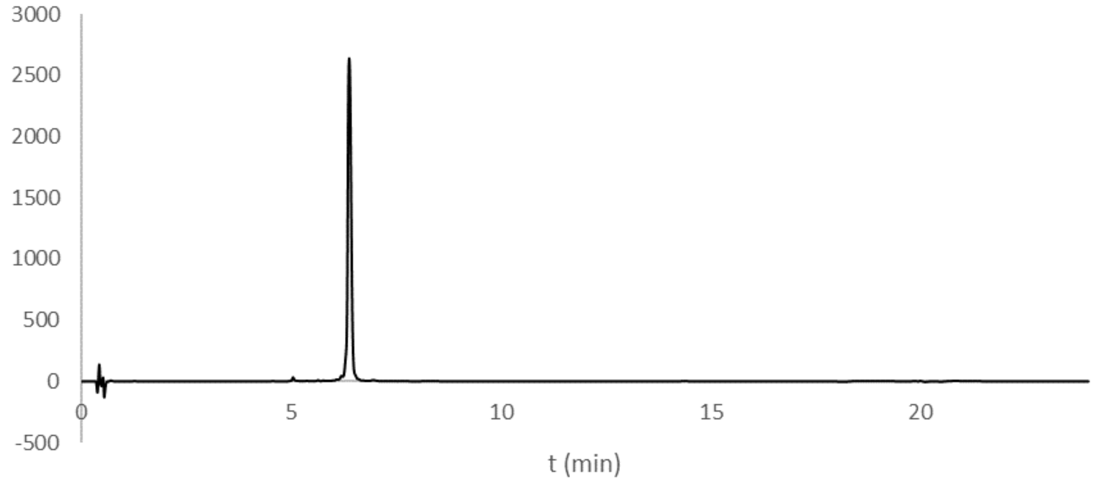

Supplemental figure 30: HPLC analysis of compound **9** (method A)

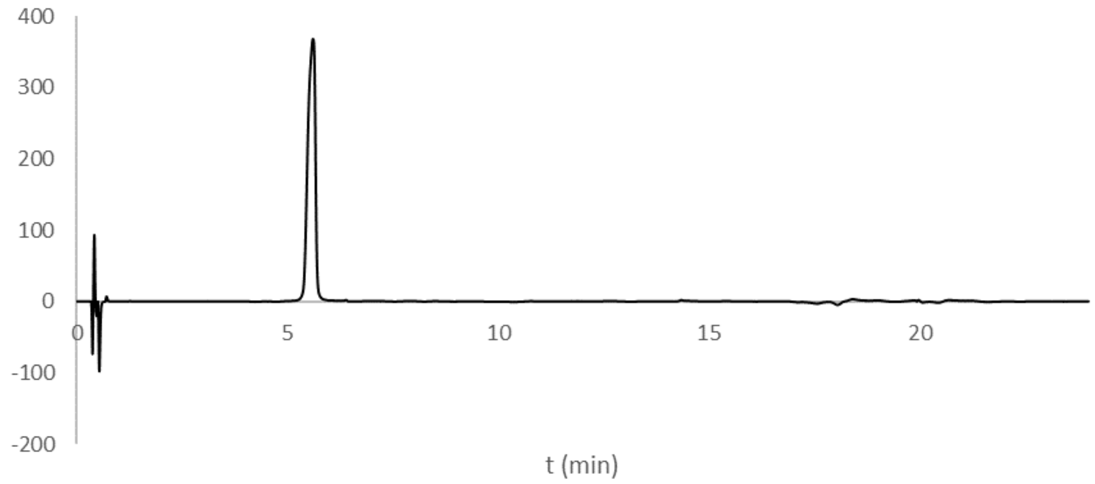

Supplemental figure 31: HPLC analysis of compound **10** (method A)

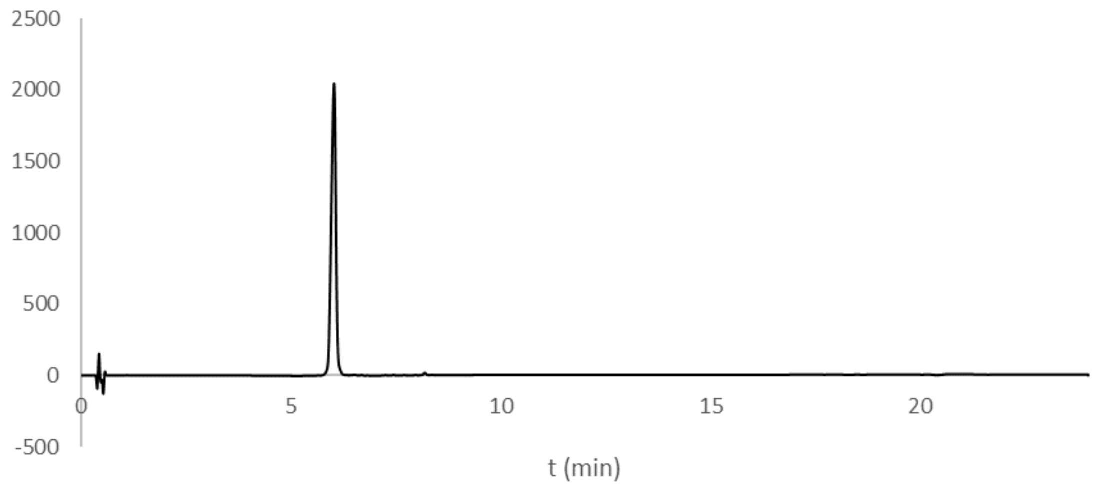

Supplemental figure 32: HPLC analysis of compound **11** (method A)

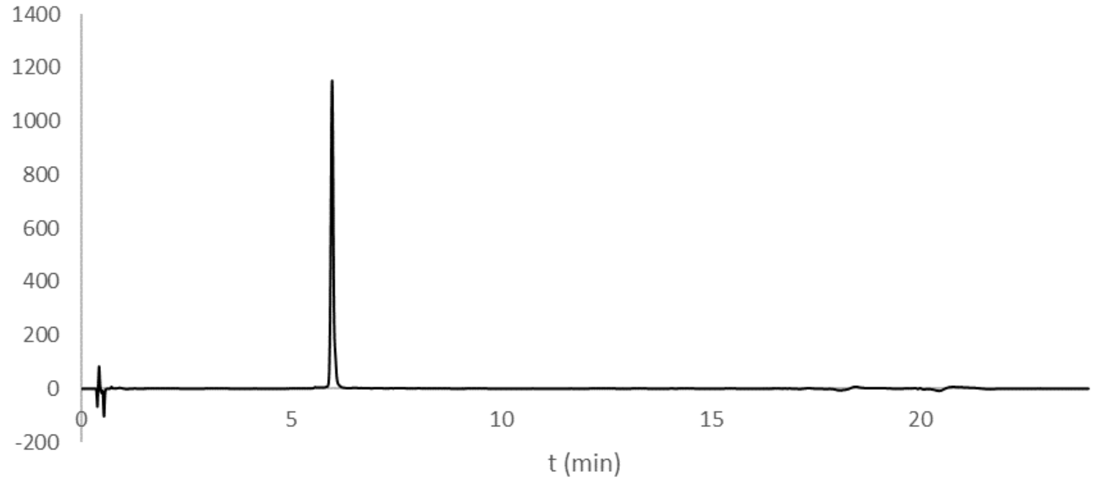

Supplemental figure 33: HPLC analysis of compound **12** (method A)

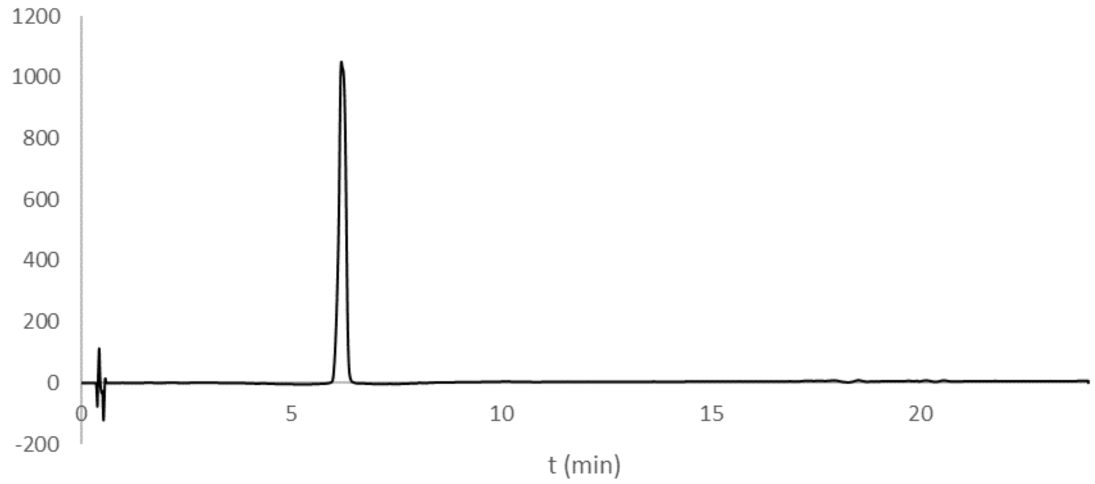

Supplemental figure 34: HPLC analysis of compound **13** (method A)

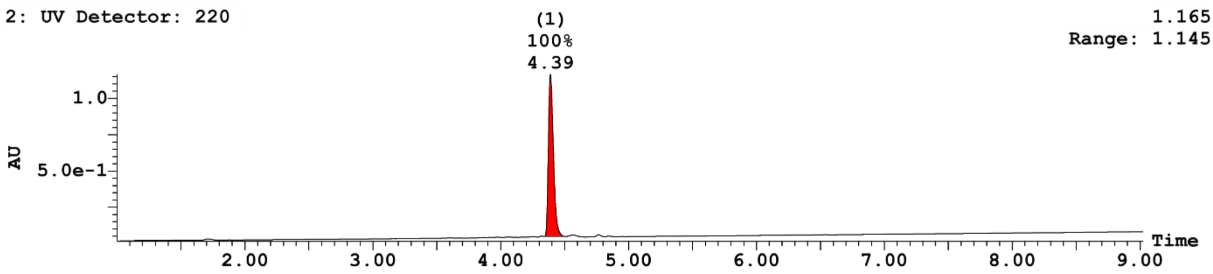

Supplemental figure 35: HPLC analysis of compound **14** (method B)

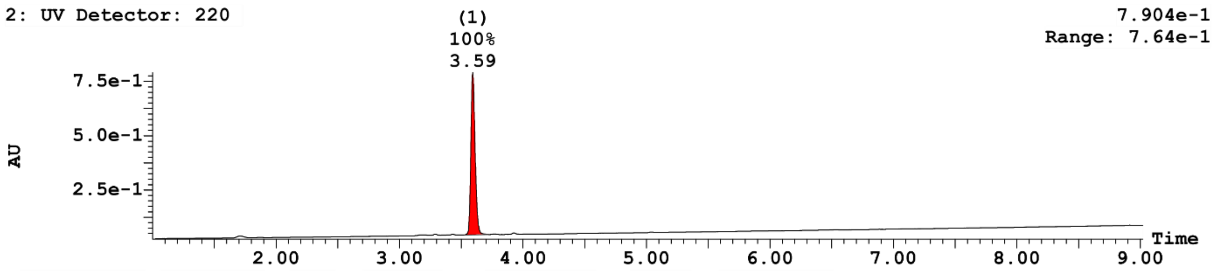

Supplemental figure 36: HPLC analysis of compound **15** (method B)

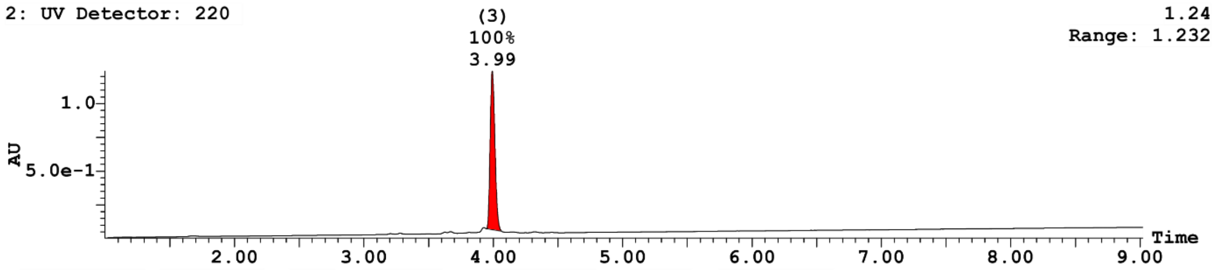

Supplemental figure 37: HPLC analysis of compound **16** (method B)

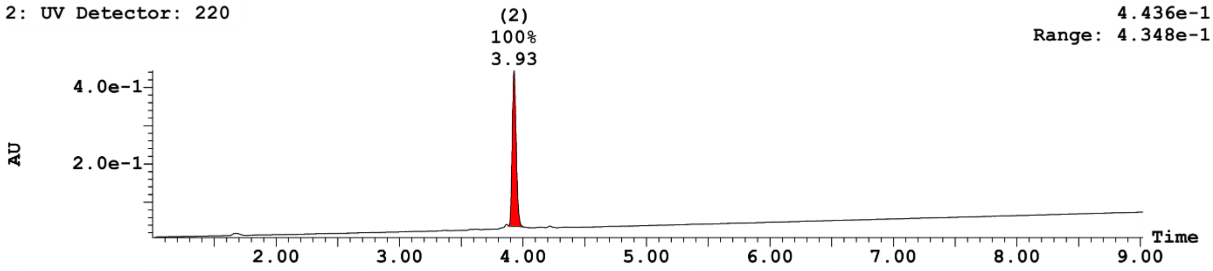

Supplemental figure 38: HPLC analysis of compound **17** (method B)

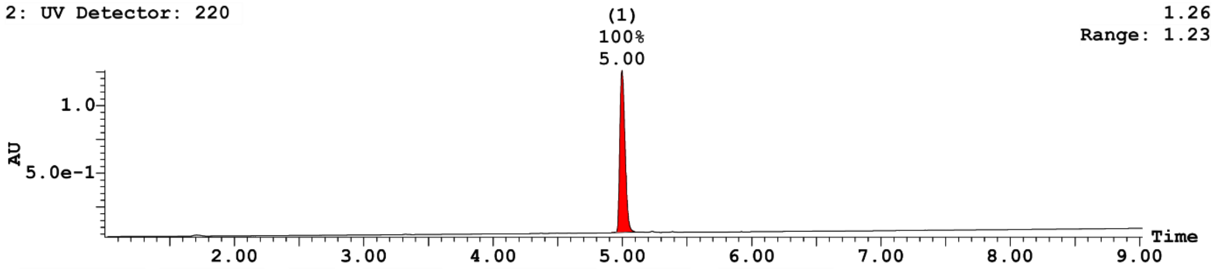

Supplemental figure 39: HPLC analysis of compound **18** (method B)

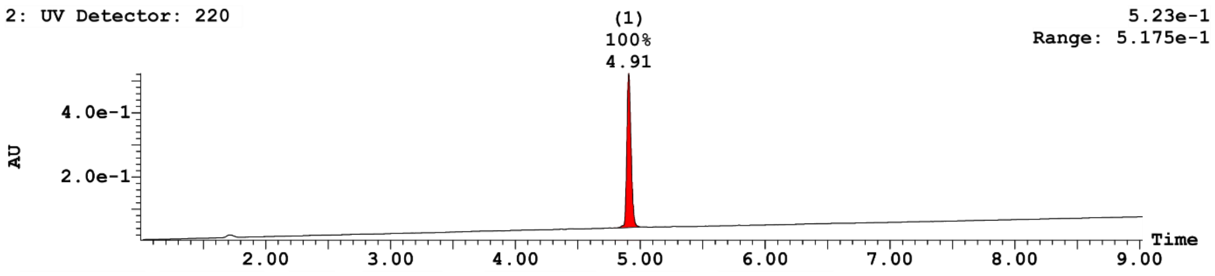

Supplemental figure 40: HPLC analysis of compound **19** (method B)

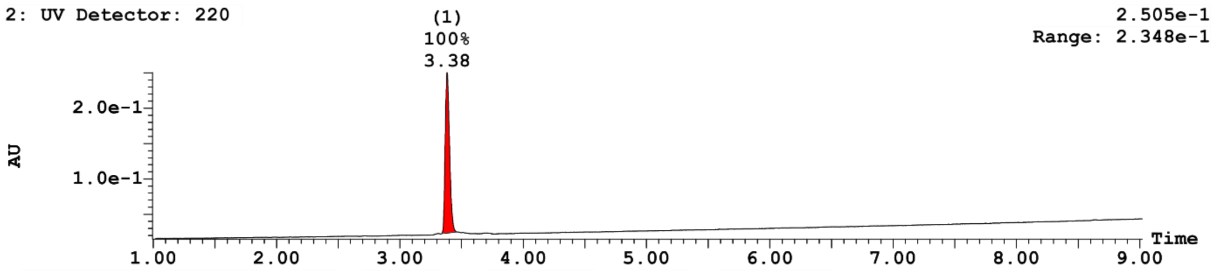

Supplemental figure 41: HPLC analysis of compound **20** (method C)

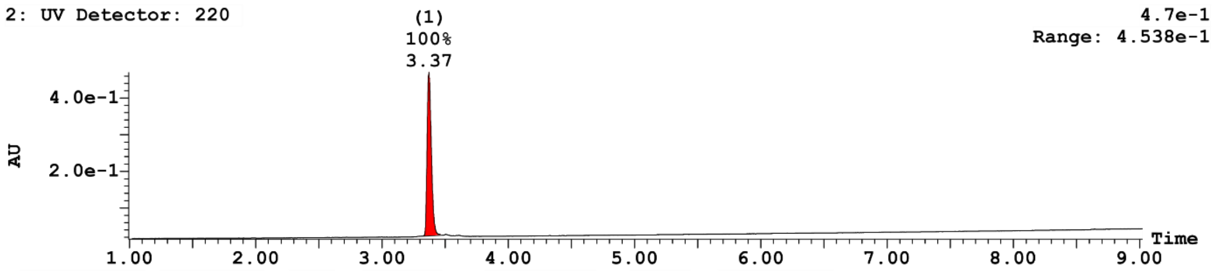

Supplemental figure 42: HPLC analysis of compound **21** (method C)

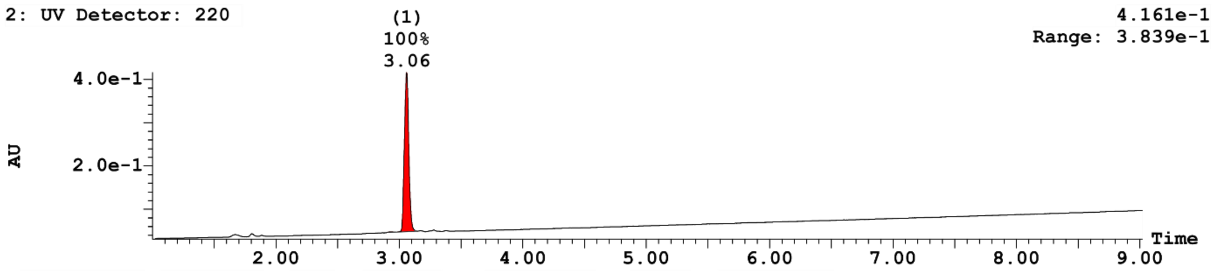

Supplemental figure 43: HPLC analysis of compound **22** (method B)

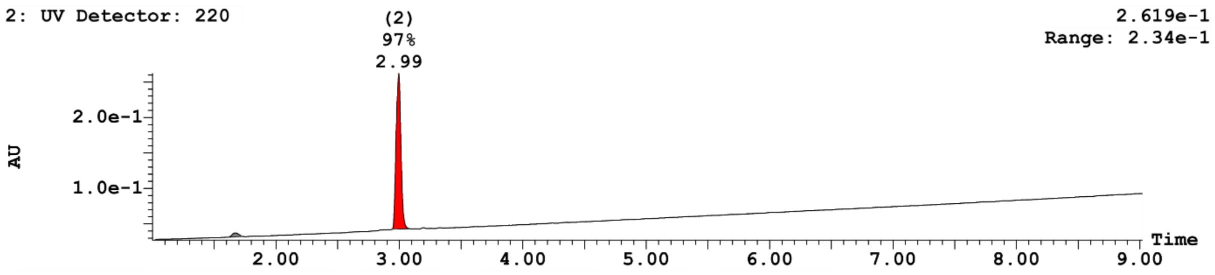

Supplemental figure 44: HPLC analysis of compound **23** (method B)

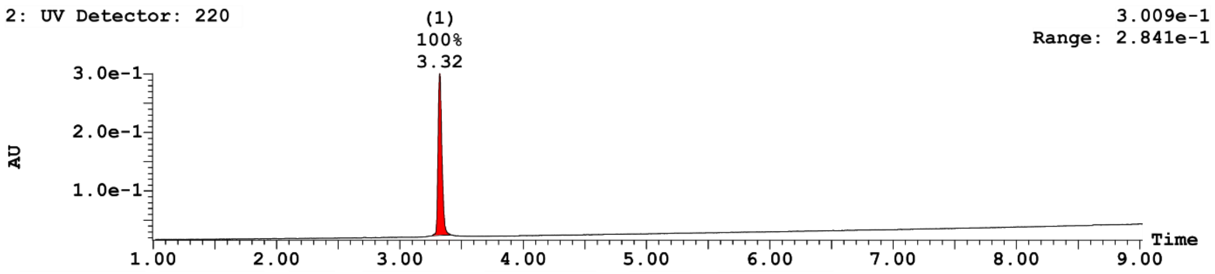

Supplemental figure 45: HPLC analysis of compound **24** (method C)

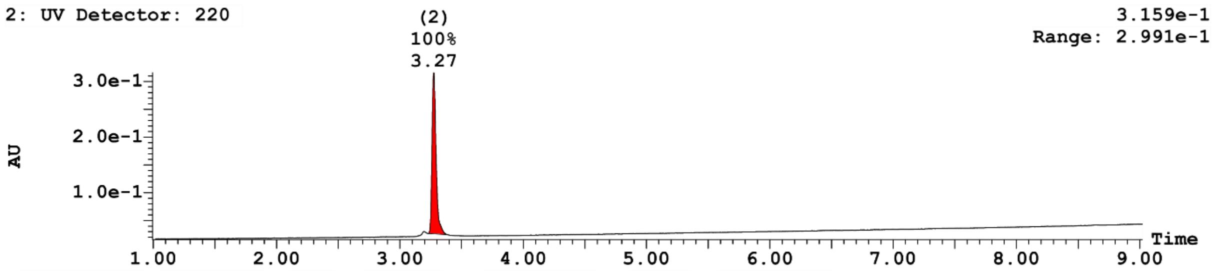

Supplemental figure 46: HPLC analysis of compound **25** (method C)

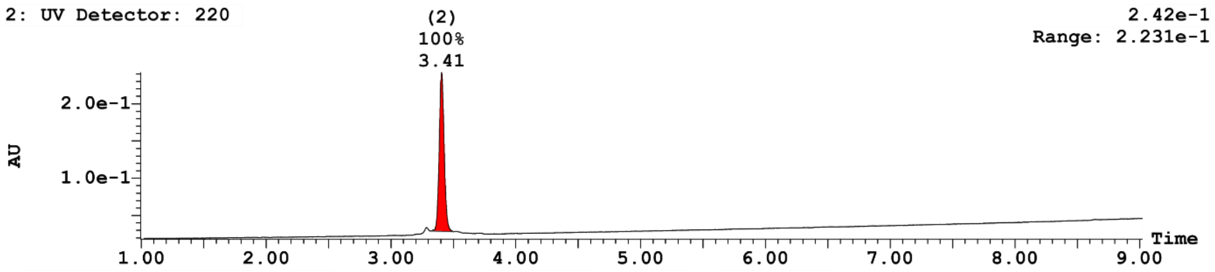

Supplemental figure 47: HPLC analysis of compound **26** (method C)

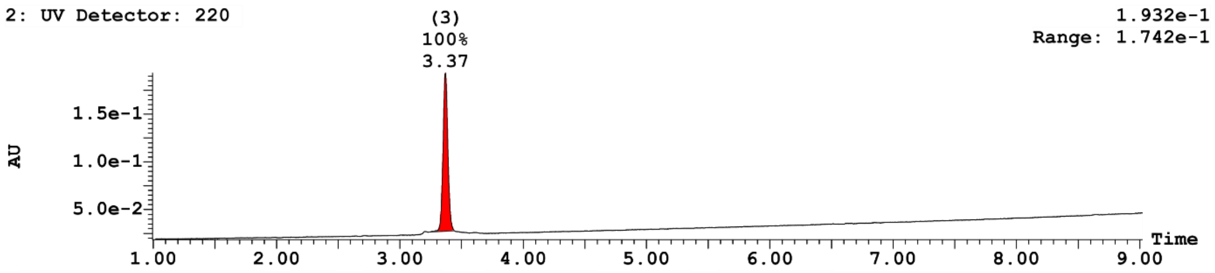

Supplemental figure 48: HPLC analysis of compound **27** (method C)

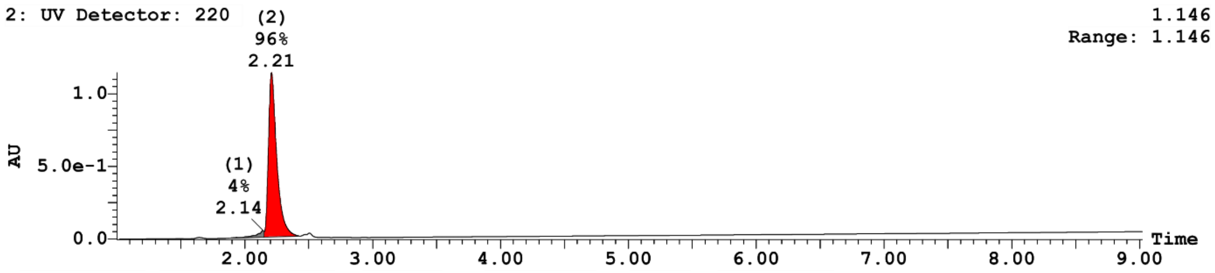

Supplemental figure 49: HPLC analysis of compound **28** (method B)

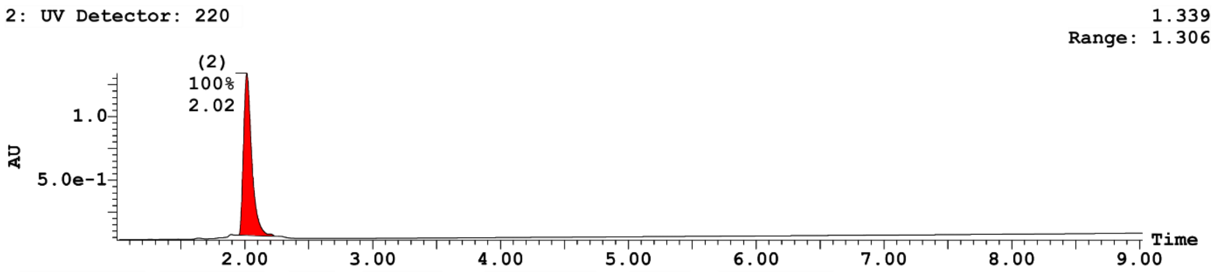

Supplemental figure 50: HPLC analysis of compound **29** (method B)

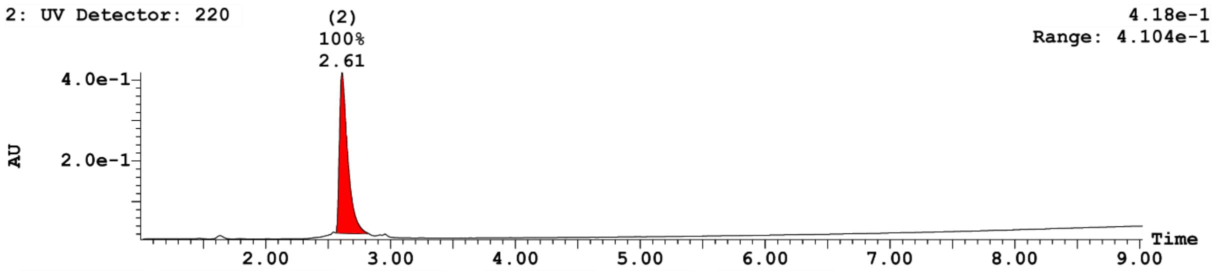

Supplemental figure 51: HPLC analysis of compound **30** (method B)

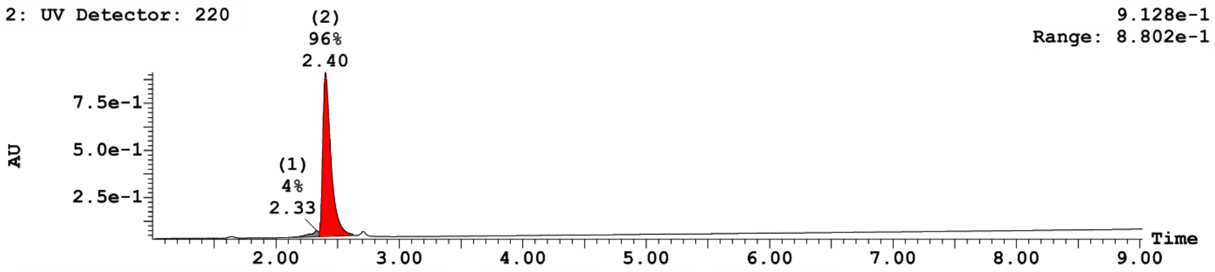

Supplemental figure 52: HPLC analysis of compound **31** (method B)

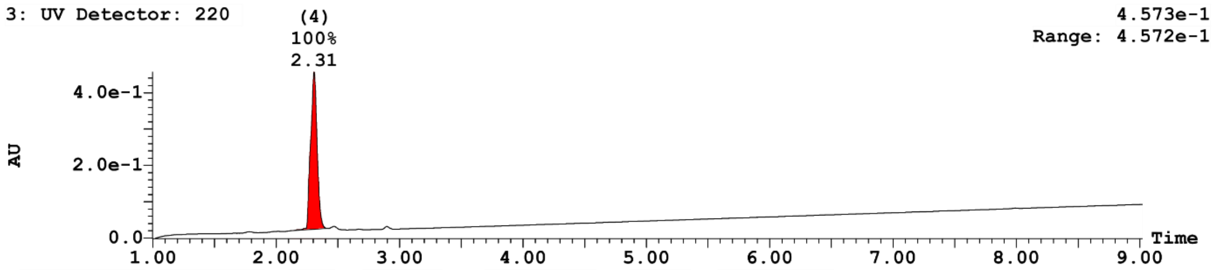

Supplemental figure 53: HPLC analysis of compound **32** (method B)

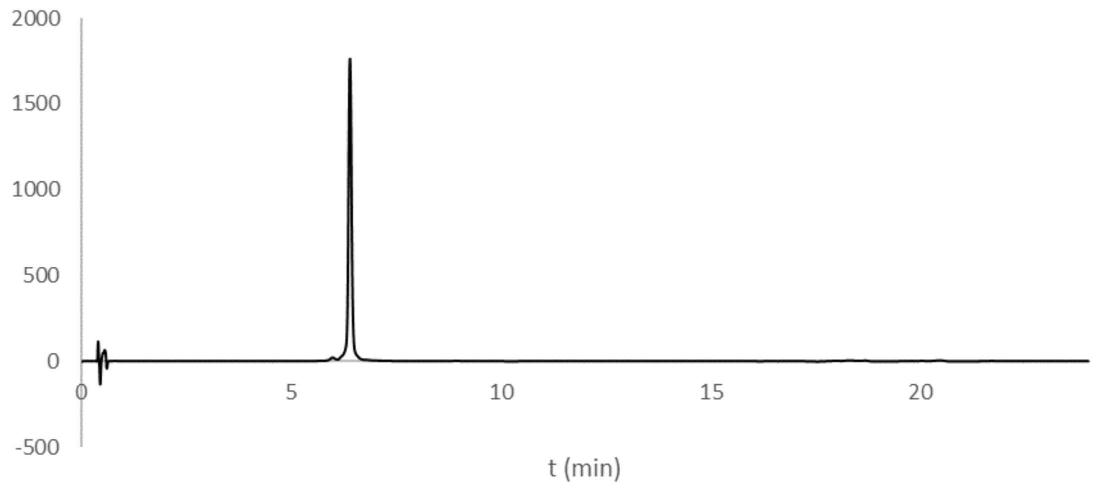

Supplemental figure 54: HPLC analysis of compound **33** (method A)

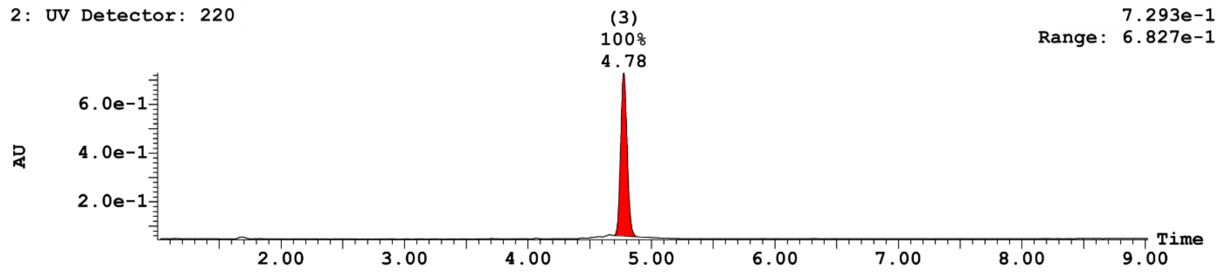

Supplemental figure 55: HPLC analysis of compound **34** (method B)

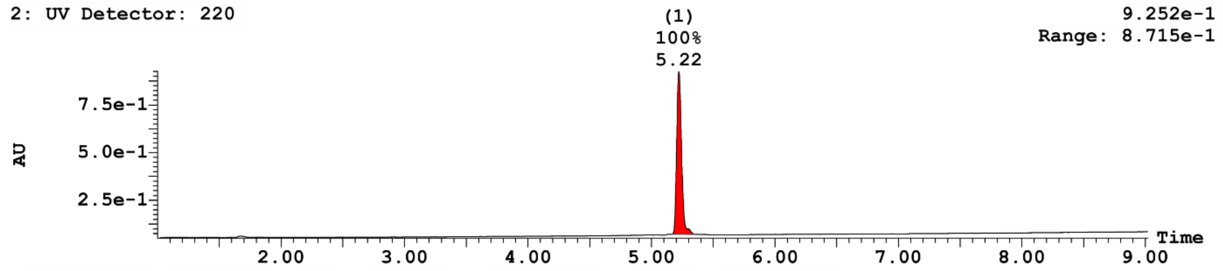

Supplemental figure 56: HPLC analysis of compound **35** (method B)

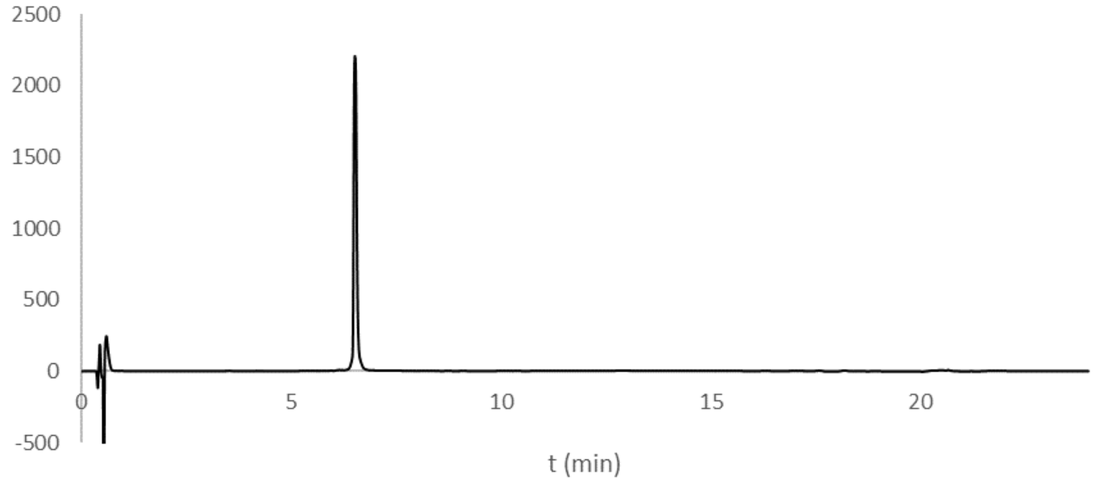

Supplemental figure 57: HPLC analysis of compound **36** (method A)

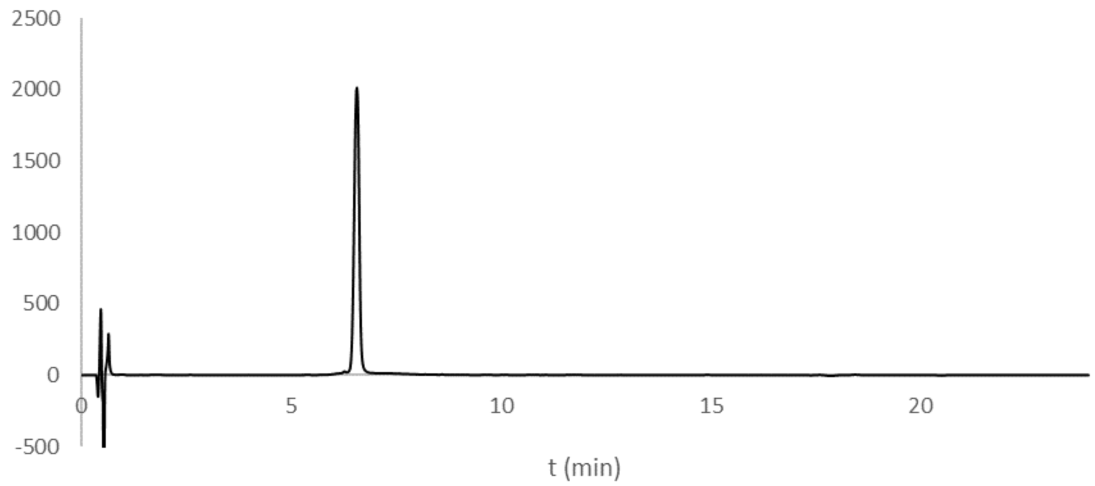

Supplemental figure 58: HPLC analysis of compound **37** (method A)

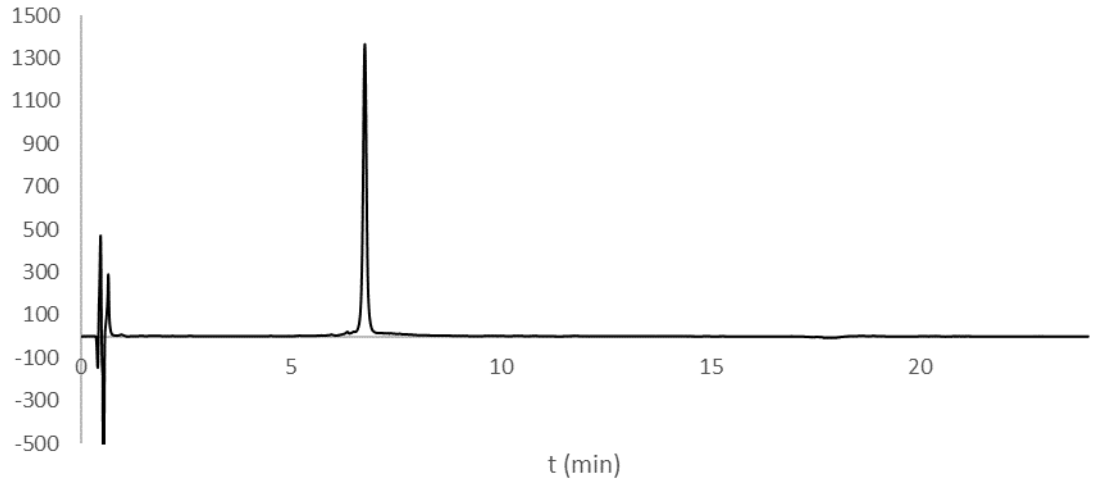

Supplemental figure 59: HPLC analysis of compound **38** (method A)

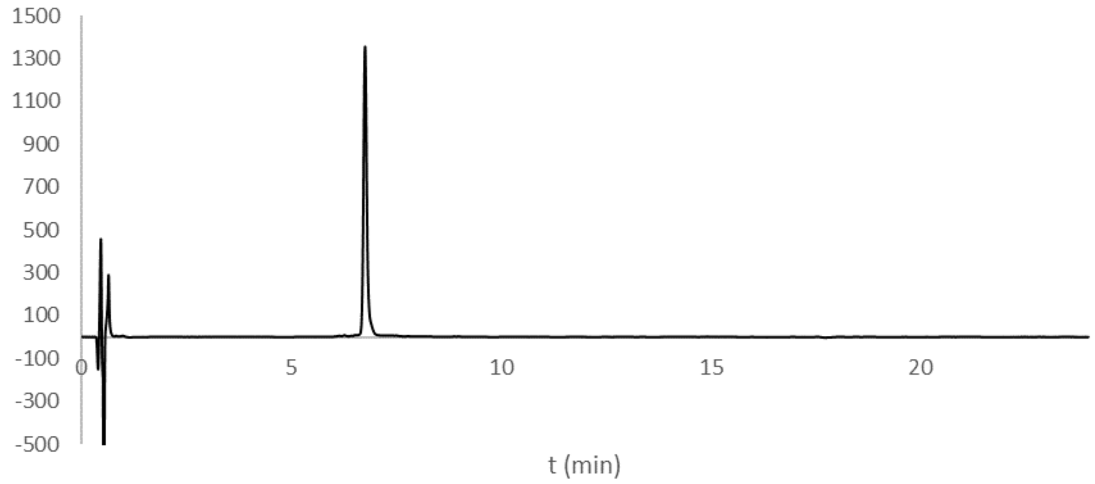

Supplemental figure 60: HPLC analysis of compound **39** (method A)

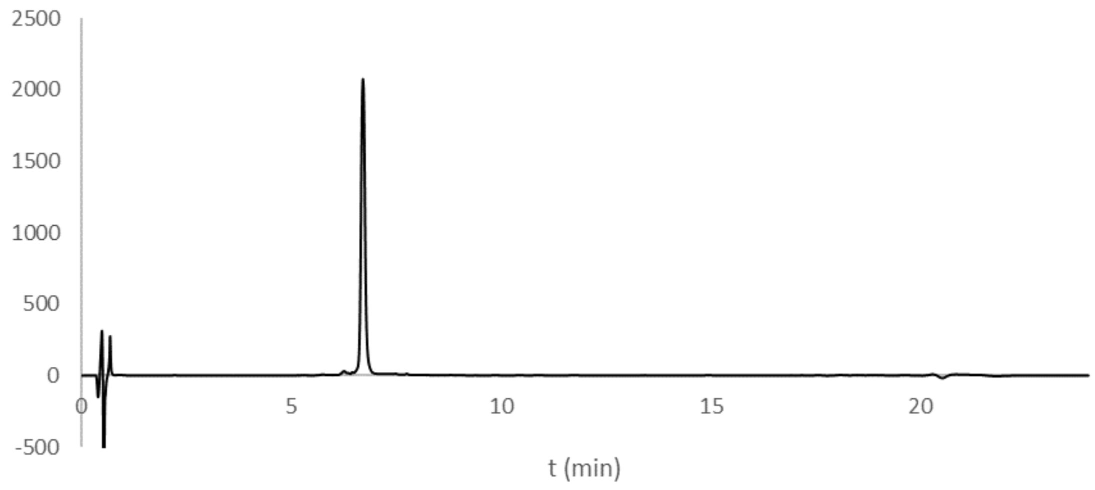

Supplemental figure 61: HPLC analysis of compound **40** (method A)

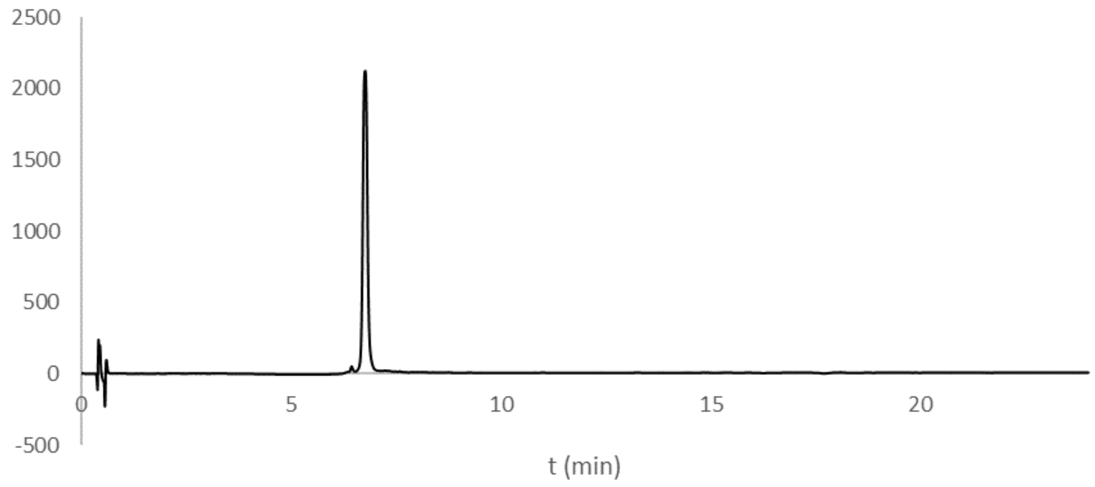

Supplemental figure 62: HPLC analysis of compound **41** (method A)

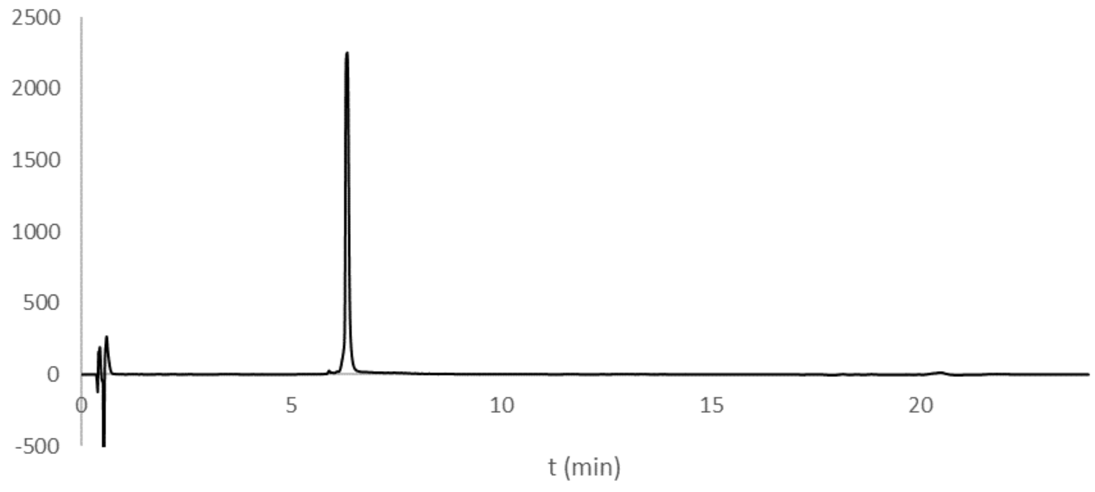

Supplemental figure 63: HPLC analysis of compound **42** (method A)

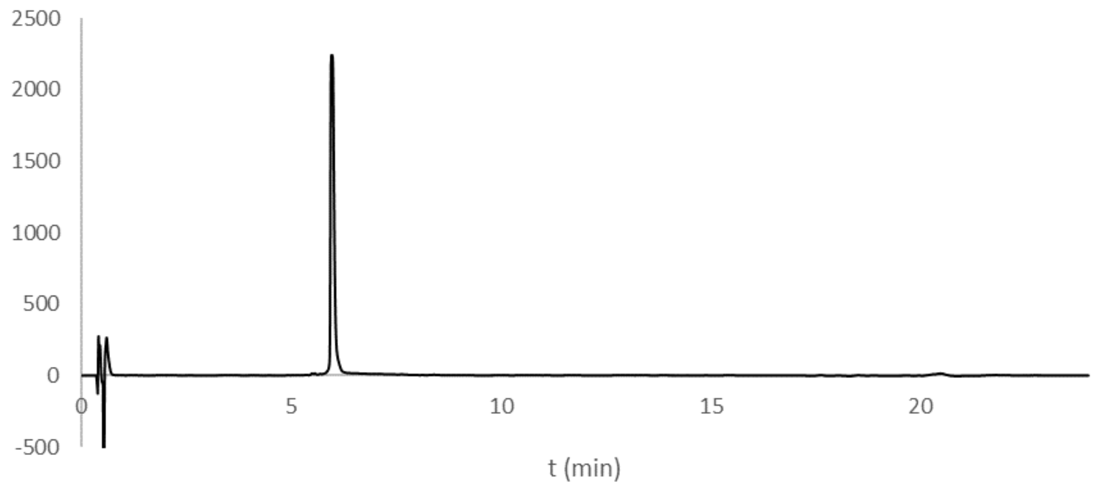

Supplemental figure 64: HPLC analysis of compound **43** (method A)

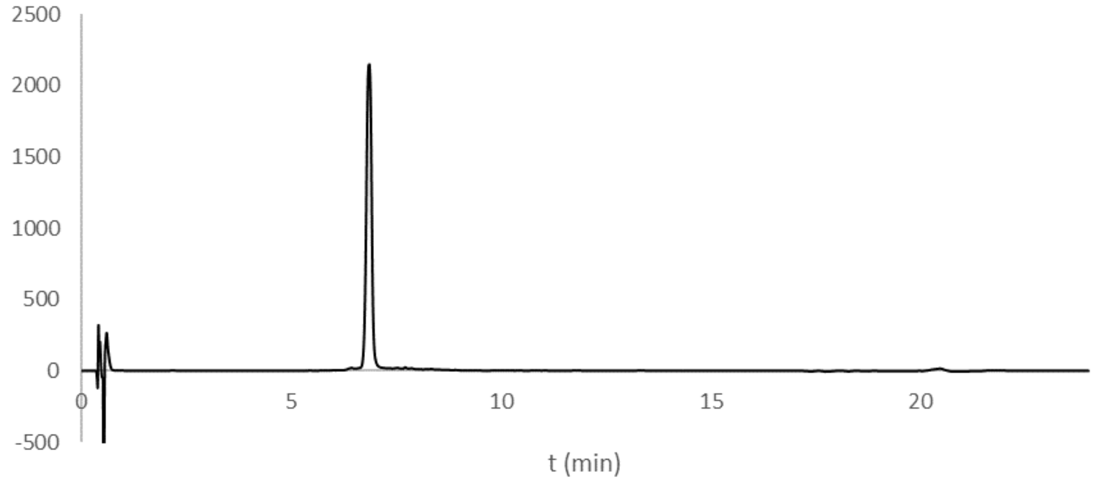

Supplemental figure 65: HPLC analysis of compound **44** (method A)

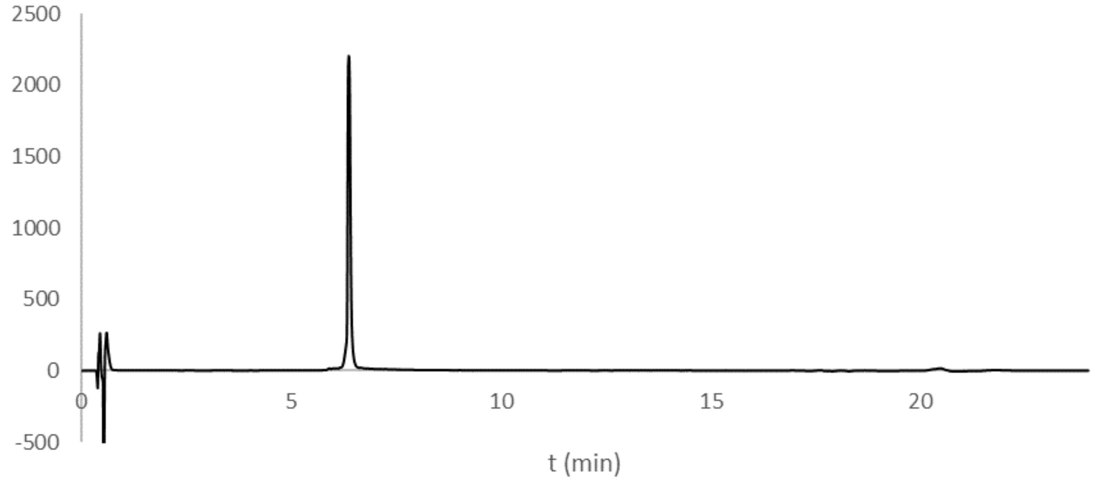

Supplemental figure 66: HPLC analysis of compound **45** (method A)

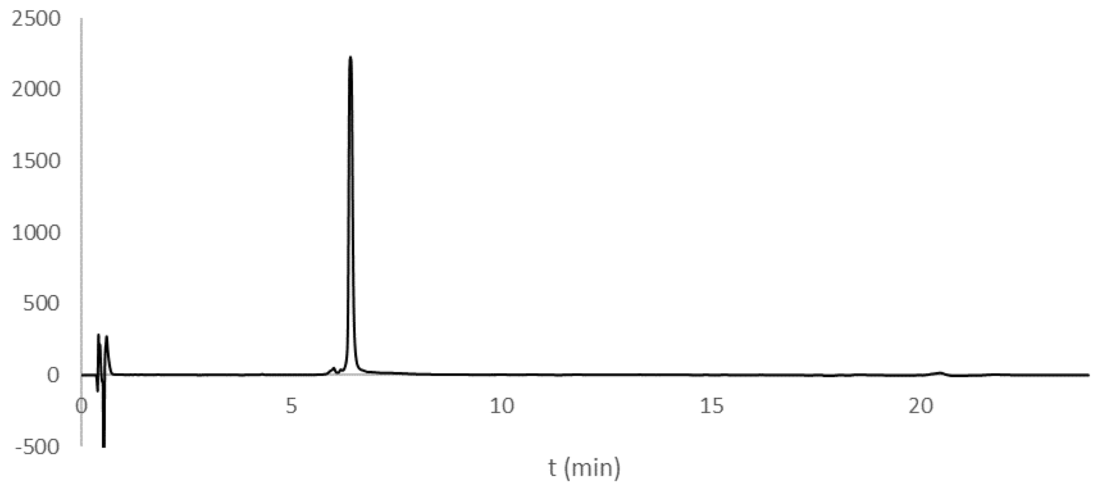

Supplemental figure 67: HPLC analysis of compound **46** (method A)

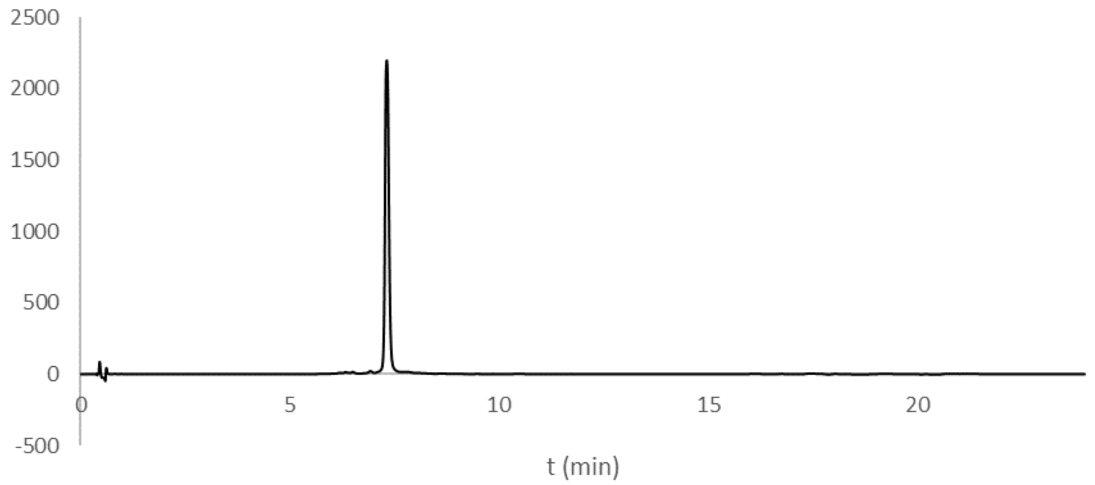

Supplemental figure 68: HPLC analysis of compound **47** (method A)

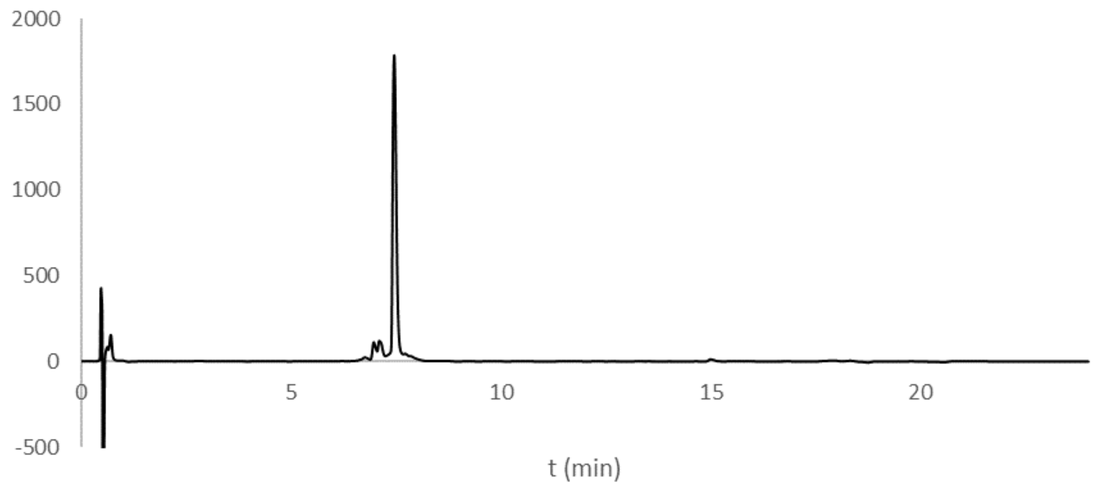

Supplemental figure 69: HPLC analysis of compound **48** (method A)

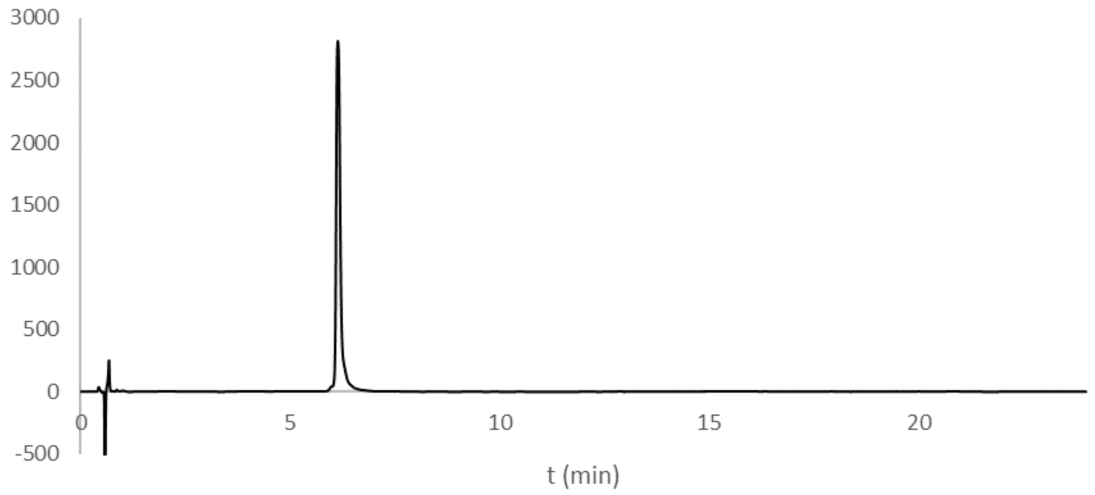

Supplemental figure 70: HPLC analysis of compound **49** (method A)

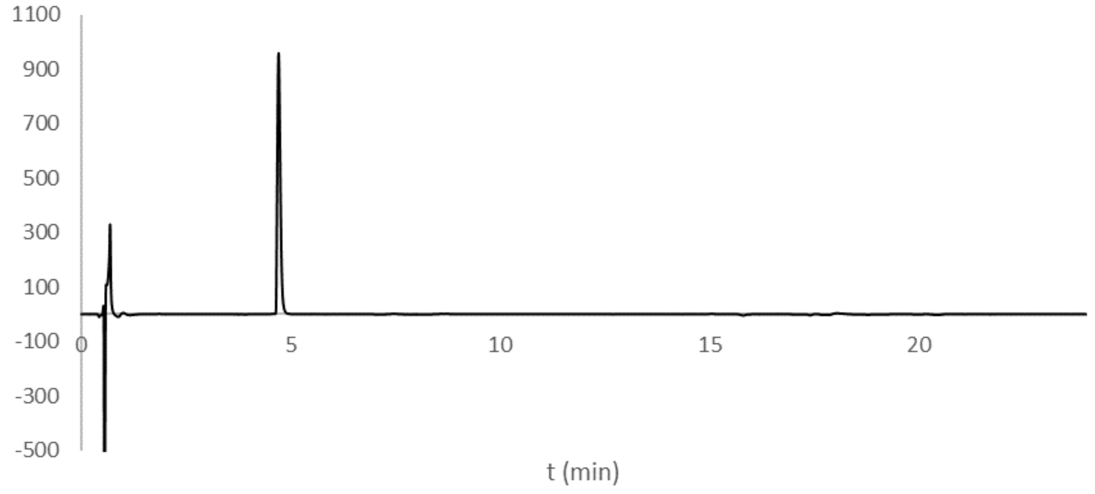

Supplemental figure 71: HPLC analysis of compound **50** (method A)

Crystallization and structure determination

5 mg/ml of full length human RBBP4 (residues 1-425, with a GP overhang on the N-terminus) was cocrystallized with a 2-fold molar excess of the macrocyclic peptides by mixing 100nl of the protein/peptide solution with 100nl reservoir at 20°C. Crystals with peptide **8** appeared in the "Classics II" screen condition H7 (Qiagen, Hilden) containing 20%w/v PEG3350 and 0.15M DL-malic acid, pH 7.1. The peptide **33** yielded thin, plate-like crystals in condition G6 of the "Classics II" screen containing 25%w/v PEG3350, 0.2M ammonium acetate and 0.1M Bis-TRIS pH 5.5. Crystals were flash-frozen in liquid nitrogen and data was collected at 100K using a Pilatus 6M detector at the X10SA beamline of SLS in Villigen, Switzerland. All data sets were integrated and scaled using XDS and XSCALE (Kabsch, 2010).

The structures were solved with PHASER (CCP4 suite) in space groups C2 (peptide **8**) and $P2_1$ (peptide **33**) by molecular replacement using chain A of RbAp48 in complex with MTA1 (PDB ID 4PBZ) as template. Both spacegroups have two molecules in the asymmetric unit and were refined using PHENIX with torsional NCS restraints, in case of peptide **8** also with TLS. The structure containing peptide **8** indexes in space group $C222_1$, but refinement in this space group did not converge and showed huge unexplained blobs in the electron density. In contrast, refinement in C2 worked very well and revealed a peculiar swap of one beta strand (residues 160 to 181) between each of the molecules in the asymmetric unit and its symmetry related molecule that is also observed in a structure of RbAp48 in complex with MTA1 (PDB ID 6G16). Residues 1 to 411 were visible in the electron density in monomer A of peptide **33**, with the loop containing residues 91 to 112 (called 5G loop since it contains 5 glycines (Millard et al, 2016)) stabilized by packing against two symmetry related molecules. In monomer B, residues 91 to 112 are disordered, similar to almost all of the related structures in the PDB except for 4PC0 (RbAp48), 4XYI (MIS16), and 6S29 (MIS16). All 5G loops are in completely different conformations, indicating that this loop is disordered in solution. In monomer A of the peptide **33** structure, residues 10-89 and 113-413 were visible, in monomer B 9-89 and 114-413.

In both structures, the peptide is involved in crystal contacts, but both xtal structures show the same binding mode of the helical part and most of the backbone as explained in the following, indicating that the binding is not significantly disturbed by crystal packing.

In the peptide **33** structure, the largest contact by far (approx. 867Å^2 in monA and 833Å^2 in monB) of the peptide **33** involves the canonical binding site where many related structures (e.g. 4PXY) have an alpha helix bound. The peptide in monomer A has a well defined electron density that shows all three cysteine links, whereas the peptide in monomer B shows a superposition of probably at least two conformations. The side chain of Phe30 in monomer B is not as parallel to the mesitylen moiety as in monomer A and is moved slightly closer to the peptide. The reason for this difference is most likely that the peptide in momomer B has extensive contacts to the N-terminal alpha helix of one and to the 5G loop (that is artificially ordered by crystal contacts) of another symmetry related molecule (440Å^2 in total), whereas the peptide in monomer A has only very few crystal contacts (81Å^2).

Compared to the linear peptide of 4PBZ, the triple link introduces some strain that slightly shifts the C-terminus of the peptide, but the main contacts to the helix and the tryptophan are undisturbed. The slightly higher affinity of the linear peptide could arise from additional contacts of the N-terminal residues of the peptide in the 4PBZ structure that are not present in the peptides **8** and **33**. Due to the additional link, the C-terminus of the cyclic peptide is pushed slightly farther away from its N-terminus so that it would clash with a symmetry related molecule in the C2 space group. This probably causes the change in space groups for the mesitylene linker peptide compared to the xylene linker.

In the peptide **8** structure, one peptide sits in the middle between the two monomers in the asymmetric unit with 887 Å^2 buried contact area to monomer A and 433 Å^2 to monomer B. The second peptide bound to monomer B buries 852 Å^2 within the main binding site, and 431 Å^2 with a symmetry-related monomer A molecule. Thus, the secondary binding site that buries the much smaller surface area is most likely a crystal packing artefact and is not seen as canonical binding site in any other structure. The main binding site has a very similar size of the contact surface area compared to the peptide **33** structure, corroborating that the two peptides bind in a similar mode. The swapped beta strand is located on the opposite side of the beta propeller compared to the main peptide binding site, so it does not influence the peptide binding mode.

The structures have been deposited with the PDB database under accession numbers 6ZRC (peptide **8**) and 6ZRD (peptide **33**).

References:

● Kabsch, 1993 W. Kabsch, Automatic processing of rotation diffraction data from crystals of initially unknown symmetry and cell constants, J. Appl. Crystallogr. 26 (1993), pp. 795

● COLLABORATIVE COMPUTATIONAL PROJECT, NUMBER 4. 1994. ``The CCP4 Suite: Programs for Protein Crystallography''. Acta Cryst. D50, 760-763.

● PHENIX: a comprehensive Python-based system for macromolecular structure solution. P.D. Adams, P.V. Afonine, G. Bunkoczi, V.B. Chen, I.W. Davis, N. Echols, J.J. Headd, L.W. Hung, G.J. Kapral, R.W. Grosse-Kunstleve, A.J. McCoy, N.W. Moriarty, R. Oeffner, R.J. Read, D.C. Richardson, J.S. Richardson, T.C. Terwilliger, and P.H. Zwart. Acta Cryst. D66, 213-221 (2010).

● Emsley, P., Lohkamp, B., Scott, W. G. & Cowtan, K. Features and development of Coot. *Acta Crystallogr D Biol Crystallogr* **66**, 486-501, doi:10.1107/S0907444910007493 (2010).

● *The PyMOL Molecular Graphics System, Version 1.6 Schrödinger, LLC.*

Supplemental Table 7: X-ray data collection and refinement statistics

	RBBP4-peptide **8** **(6ZRC)**	RBBP4-peptide **33 (6ZRD)**
Space group	C2	$P2_1$
Wavelength	0.99998	0.97852
No. xtals	1	1
synchrotron	SLS	SLS
Date	13 Oct 2017	01 Dec 2017
Detector	Pilatus 6M	Pilatus 6M
Mol/AU	2	2
a,b, c (Å)	71.96 96.33 149.3	74.4 59.95 100.22
α, β, γ (°)	90 90.014 90	90 93.826 90
Resolution (Å)	45.84-2.6 (2.67-2.6)	46.65-2.5 (2.56-2.5)
R_{sym}	11.1 (126.4)	22.70 (248.4)
$I / \sigma I$	9.91 (1.11)	7.46(0.99)
CC1/2	99.9 (82.8)	99.4(48.3)
Completeness (%)	99.5 (99.8)	99.9(100.0)
Redundancy	6.8 (7.0)	6.8(7.0)
**Refinement:**		
Resolution (Å)	45.84-2.6 (2.693-2.6)	46.65-2.5 (2.589-2.5)
No. reflections	31310 (3100)	30793 (3068)
R_{work} / R_{free}(%)	26.29/29.26	24.47/28.50
**No. atoms:**		
Protein/ Ligands	6298/36	6578/38
Water	1	7
aver. B (Å^2)	115.37	59.48
**R.m.s. deviations:**		
Bond lengths (Å)	0.011	0.007
Bond angles (°)	1.4	0.95
Clashscore	1.78	2.02
**Visible residues:**		
Chain A	10-89,113-413	1-411
Chain B	9-89, 114-413	1-90, 113-411

***** Values in parentheses are for highest resolution shell

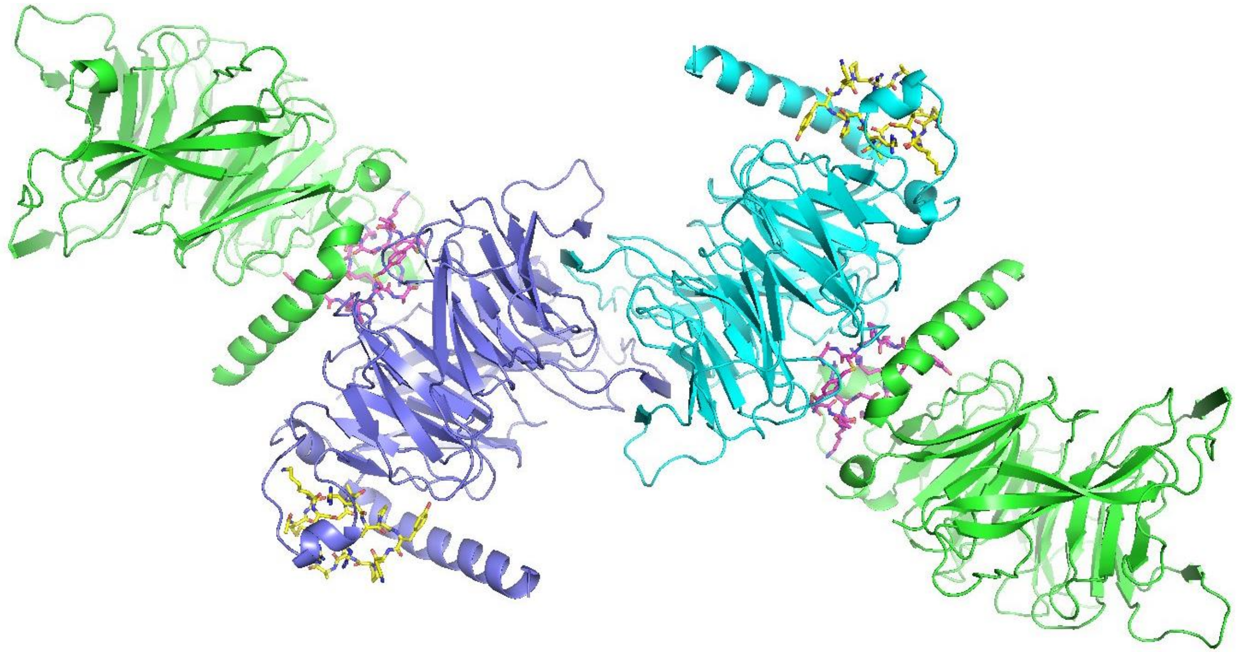

Supplemental figure 72: Beta strand swap in structure with peptide **8** – overview

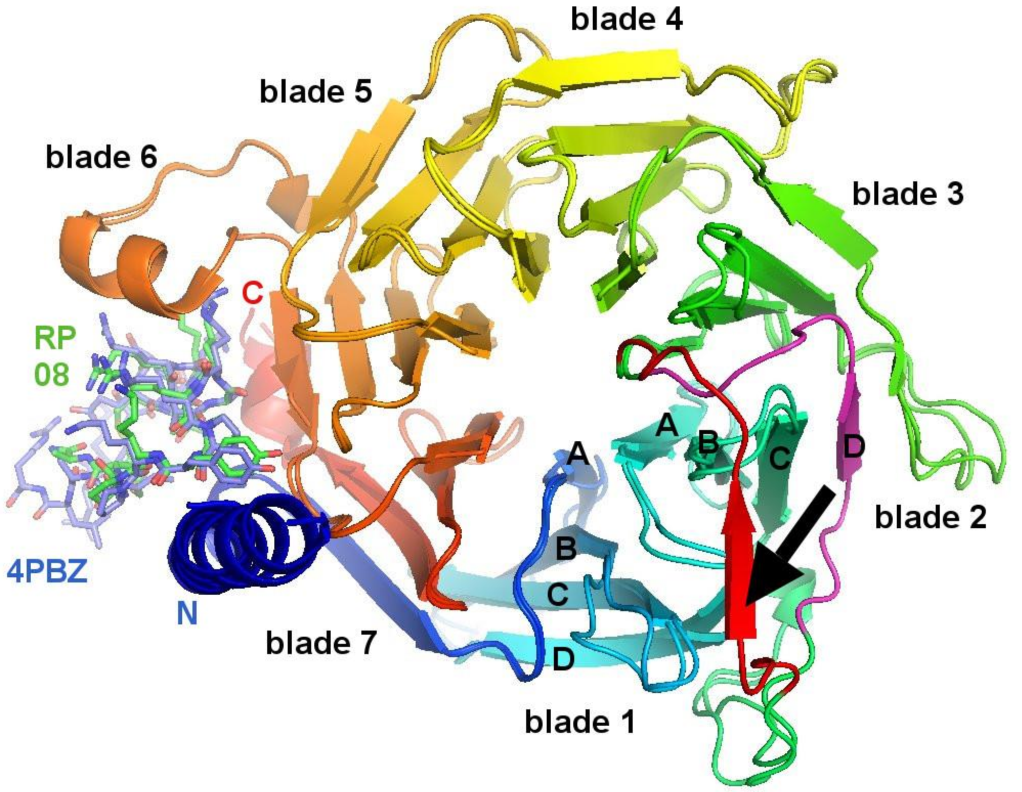

Supplemental figure 73: Beta strand swap in structure with peptide **8** – detail

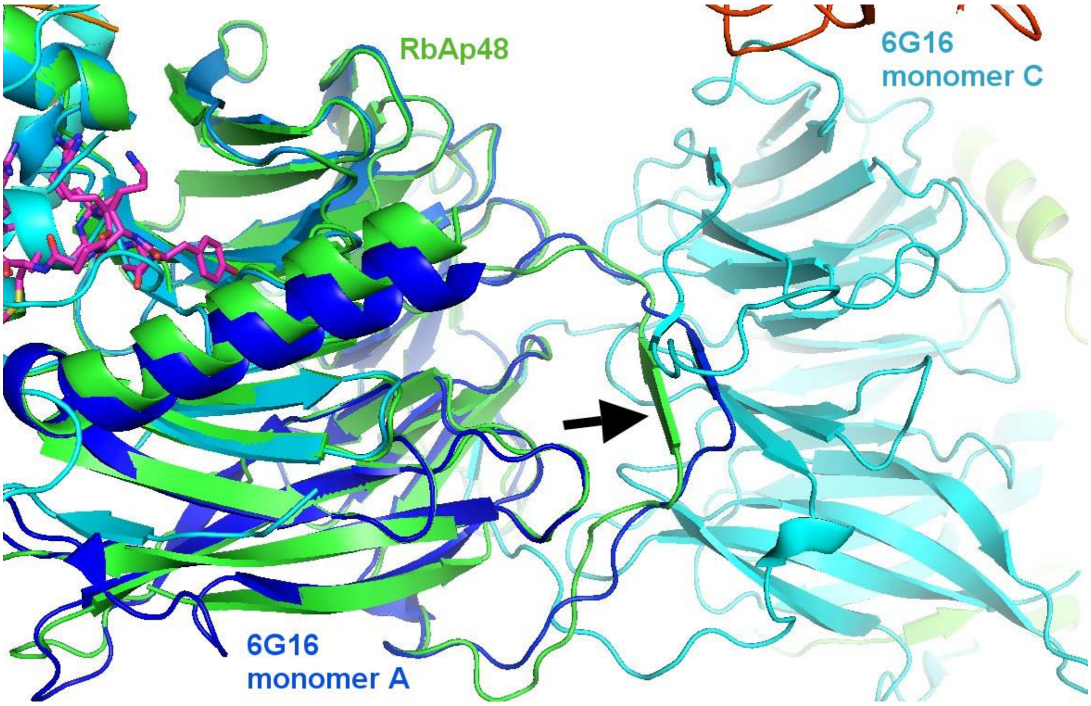

Supplemental figure 74: Beta strand swap in structure with peptide **8** compared to similar strand swap in RbAp48 (6G16)

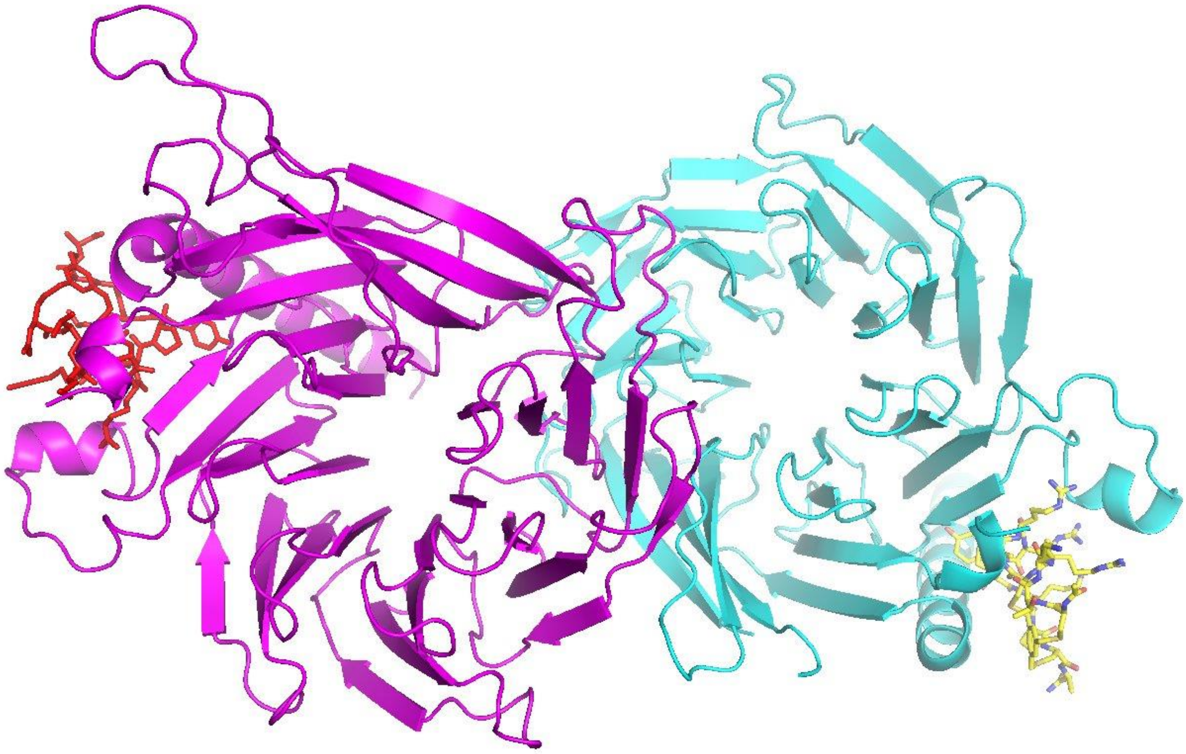

Supplemental figure 75: Asymmetric unit of RP33

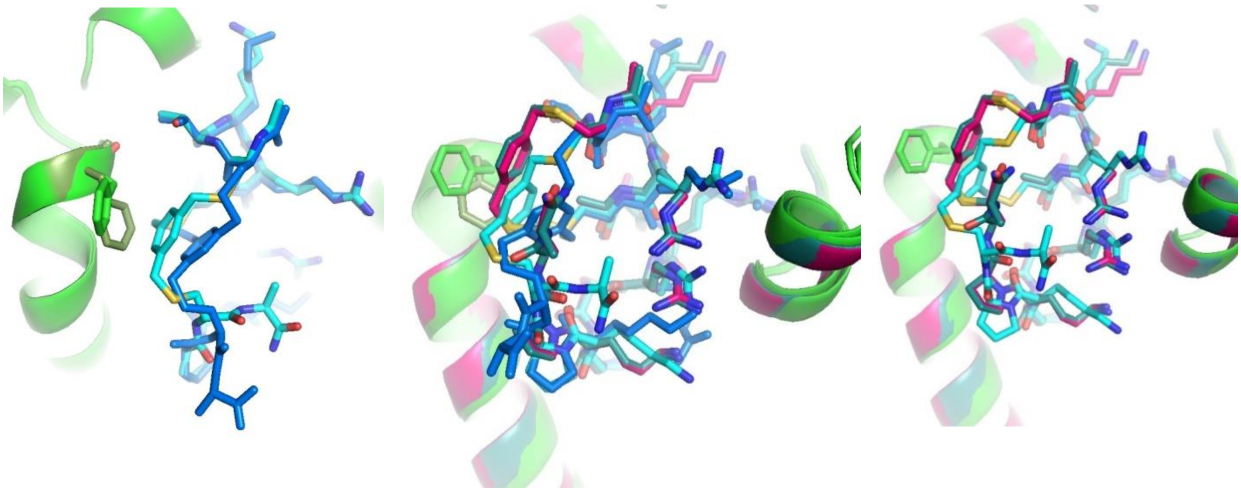

Supplemental figure 76: Structures with peptide **33** comparison Phe 30 side chain and peptide in monomers A and B (mon B dark colors)

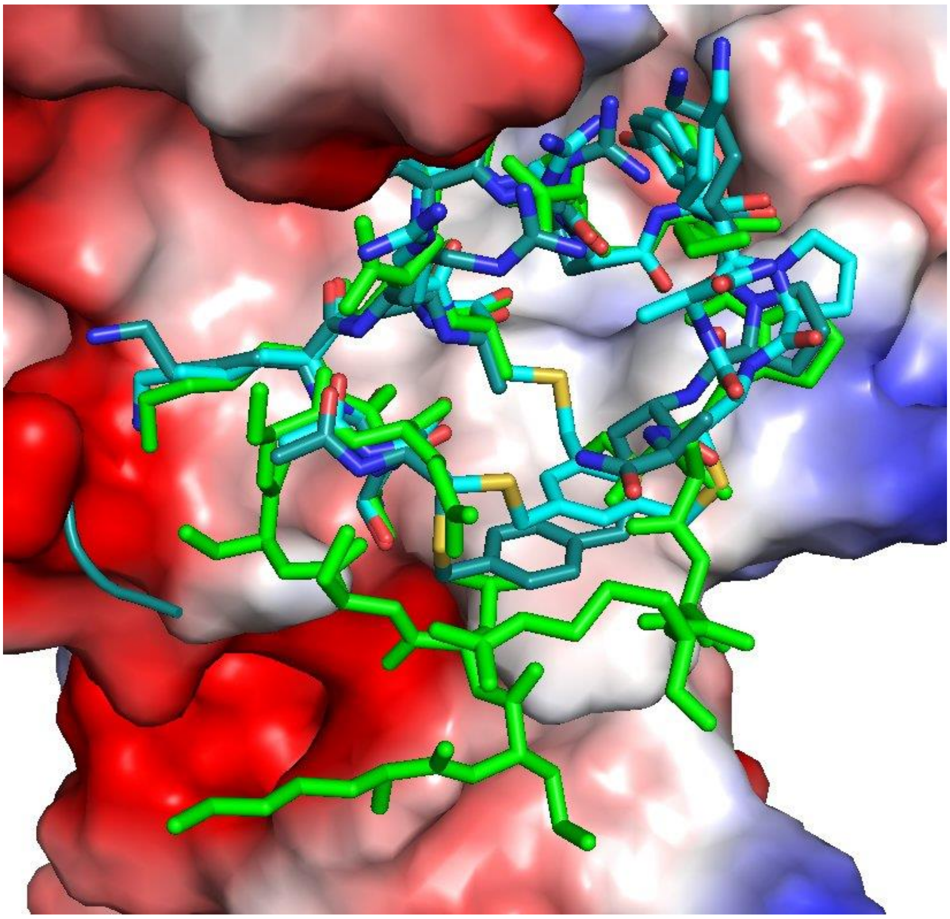

Supplemental figure 77: Comparison peptide **8** (dark blue) and peptide **33** (cyan) with open peptide of 4PBZ (green)

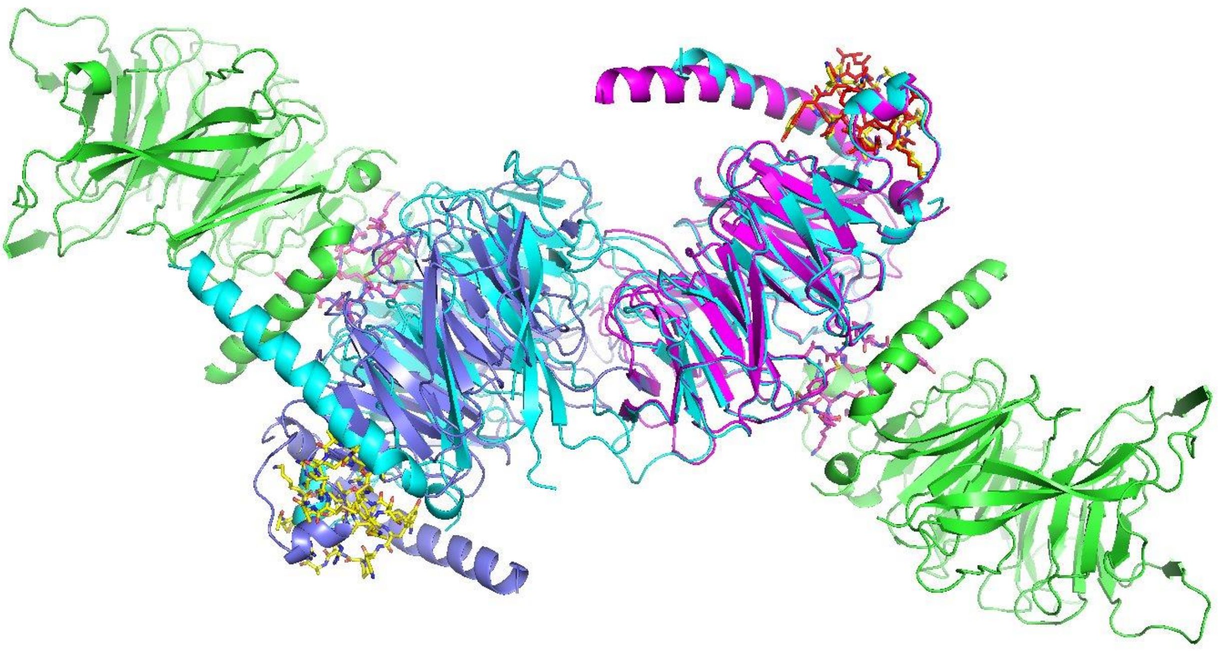

Supplemental figure 78: Compare packing of crystals with peptide **8** and peptide **33**
